# Short turnaround time of seven to nine hours from sample collection until informed decision for sepsis treatment using nanopore sequencing

**DOI:** 10.1038/s41598-024-55635-z

**Published:** 2024-03-19

**Authors:** Jawad Ali, Wenche Johansen, Rafi Ahmad

**Affiliations:** 1 Department of Biotechnology, Inland Norway University of Applied Sciences, Holsetgata 22, 2317 Hamar, Norway; 2 Institute of Clinical Medicine, Faculty of Health Sciences, UiT - The Arctic University of Norway, Hansine Hansens Veg 18, 9019 Tromsø, Norway

**Keywords:** Infectious-disease diagnostics, Genome informatics

## Abstract

Bloodstream infections (BSIs) and sepsis are major health problems, annually claiming millions of lives. Traditional blood culture techniques, employed to identify sepsis-causing pathogens and assess antibiotic susceptibility, usually take 2–4 days. Early and accurate antibiotic prescription is vital in sepsis to mitigate mortality and antibiotic resistance. This study aimed to reduce the wait time for sepsis diagnosis by employing shorter blood culture incubation times for BD BACTEC™ bottles using standard laboratory incubators, followed by real-time nanopore sequencing and data analysis. The method was tested on nine blood samples spiked with clinical isolates from the six most prevalent sepsis-causing pathogens. The results showed that pathogen identification was possible at as low as 10^2^–10^4^ CFU/mL, achieved after just 2 h of incubation and within 40 min of nanopore sequencing. Moreover, all the antimicrobial resistance genes were identified at 10^3^–10^7^ CFU/mL, achieved after incubation for 5 h and only 10 min to 3 h of sequencing. Therefore, the total turnaround time from sample collection to the information required for an informed decision on the right antibiotic treatment was between 7 and 9 h. These results hold significant promise for better clinical management of sepsis compared with current culture-based methods.

## Introduction

Bloodstream infections (BSIs) and sepsis are major health problems and are responsible for millions of deaths each year across the globe. A recent study in 2017 estimated 48.9 million cases and 11 million sepsis-related deaths worldwide, accounting for almost 20% of all global deaths^1^. Around 85% of these cases and deaths occurred in low- and middle-income countries. Sepsis is a significant cause of maternal, neonatal, and child mortality, with half of all global sepsis cases occurring among children^1^. Therefore, combating sepsis will contribute to achieving the United Nations Sustainable Development Goal 3, ensuring healthy lives and promoting well-being for all ages.

Sepsis occurs when pathogens (most commonly bacteria) enter the bloodstream, and the body's immune system responds to the infection, causing damage or even failure of the host's tissues and organs^2,3^. Some of the most common sepsis-causing bacteria include *Staphylococcus aureus* (*S. aureus*), *Escherichia coli* (*E. coli*), *Klebsiella pneumoniae* (*K. pneumoniae*), *Pseudomonas aeruginosa* (*P. aeruginosa*), and *Enterococcus faecalis* (*E. faecalis*)^4^. Moreover, *Acinetobacter* spp. has been detected in neonatal sepsis^5,6^. Sepsis is considered a medical emergency. It has been reported that with each hour of delay in initiating treatment, the patient's survival chances decrease by 7.6%^7^. Moreover, it was reported that a delay in antibiotic administration beyond 1 h in children with sepsis was associated with higher mortality^8^. Therefore, starting antibiotic therapy as early as possible is crucial to increase the chances of the patient's survival. The guidelines for treating sepsis propose starting antibiotic treatment within 1 h of the onset of symptoms^9^. Most early antibiotics prescribed for treating sepsis are empirical and broad-spectrum. Identifying the bacterial pathogen is time-consuming, and the physicians must specify the empiric therapy. In some cases, this empirical antibiotic therapy may be effective and increase the chances of patient survival^10^. Still, it might also contribute to the global problem of antimicrobial resistance (AMR)^11^. A study performed in 2015 reviewing the scientific literature (2004 to 2014) reported inappropriate empirical antibiotic treatment in severe infections from 14 to 79% of the cases^12^. In June 2023, the World Health Organization (WHO) published its global research agenda for AMR in human health. Diagnosing pathogens and their antimicrobial susceptibility testing directly from positive blood culture bottles has been highlighted as a research priority in the policy brief^13^.

Traditional blood culturing is the current gold standard for sepsis diagnosis. The routine blood cultures take approximately 24–72 h of incubation to become positive due to the low concentration of microbial cells in the bloodstream (usually 1–100 colony-forming units (CFU) per mL) combined with a slow pathogen growth rate^14–16^. In addition, it takes several days to identify the pathogens and their sensitivity and resistance to antibiotics, which can delay the treatment with the appropriate drug even further^3,17^. Also, a higher blood volume is required to perform blood culturing replicates, which is challenging to obtain, especially in the case of children and neonates^18,19^. In addition to routine biochemical approaches, there are additional methods for the detection of pathogens, including matrix-assisted laser desorption/ionization-time of flight mass spectrometry (MALDI-TOF MS), polymerase chain reaction (PCR), and fluorescence in situ hybridization (FISH). However, each technology has some limitations regarding the rapid identification of pathogens and their associated antibiotic resistance genes (ARGs), detailed in a recent review by Avershina et al.^20^.

The potential of nanopore sequencing in clinical microbiology for identifying pathogens and ARGs for outbreak surveillance has been shown in many studies^21–25^. Nanopore sequencing is mainly performed using a MinION device (Oxford Nanopore Technologies, United Kingdom). This small pocketable sequencing device can be connected to a computer through USB and provide sequencing data that can be analyzed in real-time^17,26–28^. Some of the advantages offered by nanopore sequencing are short library preparation time, long sequencing reads, and a wide variety of sequencing kits to choose from depending on the target sample^29^. We previously showed that nanopore sequencing could detect bacterial pathogens from positive blood cultures within 10 min and ARGs and plasmids within 1 h of sequencing^28^. Similarly, using Flongle flow cells, we have shown that detecting pathogens and their corresponding AMR encoding genes was possible within 10 min and 3 h of sequencing, respectively^16^. In the same study, we showed that the overall time from sample collection to information on pathogen and AMR profile was > 24 h, which included the time for culture being tagged positive by the automated blood culture incubation system, DNA extraction, library preparation, and nanopore sequencing.

The work presented here aimed to investigate the limit of detection by employing shorter culture incubation time followed by nanopore sequencing (including both MinION and Flongle flow cells), thus reducing the wait time for sepsis diagnosis compared to traditional methods. To achieve this goal, healthy and fresh blood samples were spiked with sepsis-relevant initial inocula of nine clinical bacterial isolates, which were incubated in BD BACTEC™ blood culture bottles, followed by nanopore sequencing and subsequent detection of the pathogen and ARGs. The results show that the most common sepsis-causing pathogens and their ARGs could be successfully identified after only 2 to 5 h of incubation using a standard laboratory incubator and nanopore sequencing. The overall Turn-Around-Time (TAT) for identifying bacteria and ARGs, including incubation of the cultures, DNA extraction, library preparation, sequencing, and data analysis, was between 7 and 9 h. Such a workflow that combines shorter incubation time followed by real-time nanopore sequencing and data analysis could be transformative for detecting and identifying infections and AMR in human health.

## Results

### Growth characteristics of sepsis-relevant bacteria in BD BACTEC blood culture medium grown in a standard incubator

Samples from the blood cultures were harvested every 3 h of incubation for up to 12 h, with subsequent quantification of CFU/mL at each time point. During the incubation, an apparent change in the color of the blood culture was detected, evolving from its original fresh human blood color to darker red as the number of CFU/mL increased (data not shown). This observed color alteration holds promise as a visual indicator for detecting bacterial growth in blood cultures.

Out of the nine bacterial strains analyzed, four bacterial strains, *E. coli* CCUG17620, *A. baumannii* CCUG19096T, *E. faecalis* CCUG9997, and *K. pneumoniae* 225, showed an increase in CFU/mL after 3 h of incubation to 40, 160 and 20 CFU/mL, respectively. The other tested bacteria either did not grow after 3 h of incubation or the number of CFU/mL was under the detection limit of the method used. The bacterial isolates showed growth proportional to the incubation time. The results showed that, for the majority of the cultures, CFU/mL reached 10^3^ after 6 h of incubation except for *S. aureus* NCTC8325, *E. faecalis* CCUG9997, and *P. aeruginosa* CCUG17619, where the CFU/mL was 10^4^ and 10^2^, respectively (Table 1).

**Table 1:** An overview of bacterial isolates, CFU/mL at different time points, time '0', and DNA concentration of the samples sequenced using MinION.

Nr	Bacteria	6 h incubation		9 h incubation		12 h incubation		CFU/mL at "t_0_"
		CFU/mL	DNA ng/µL	CFU/mL	DNA ng/µL	CFU/mL	DNA ng/µL	
1	*E. coli* CCUG17620	6.6 × 10^3^	97.1	5.7 × 10^6^	110	2.1 × 10^9^	110	250
2	*E. coli* NCTC13441	1.9 × 10^3^	67.4	2.6 × 10^6^	110	3.5 × 10^8^	110	15
3	*S. aureus* NCTC8325	1.2 × 10^4^	100	5 × 10^6^	100	3.1 × 10^7^	100	30
4	*S. aureus* CCUG35600	4.2 × 10^3^	100	1.1 × 10^5^	110	4.9 × 10^6^	100	20
5	*K. pneumoniae* CCUG225T	1.2 × 10^3^	100	2 × 10^6^	100	1.9 × 10^7^	110	35
6	*K. pneumoniae* 225	4.4 × 10^3^	55.3	1.1 × 10^7^	100	1 × 10^9^	110	130
7	*A. baumannii* CCUG19096T	1.6 × 10^3^	100	1.6 × 10^4^	100	2.4 × 10^7^	97	90
8	*P. aeruginosa* CCUG17619	1.8 × 10^2^	110	9 × 10^3^	110	3 × 10^6^	97	06
9	*E. faecalis* CCUG9997	4 × 10^4^	74.8	1 × 10^7^	116	1.2 × 10^9^	120	1550

**Figure 1:**
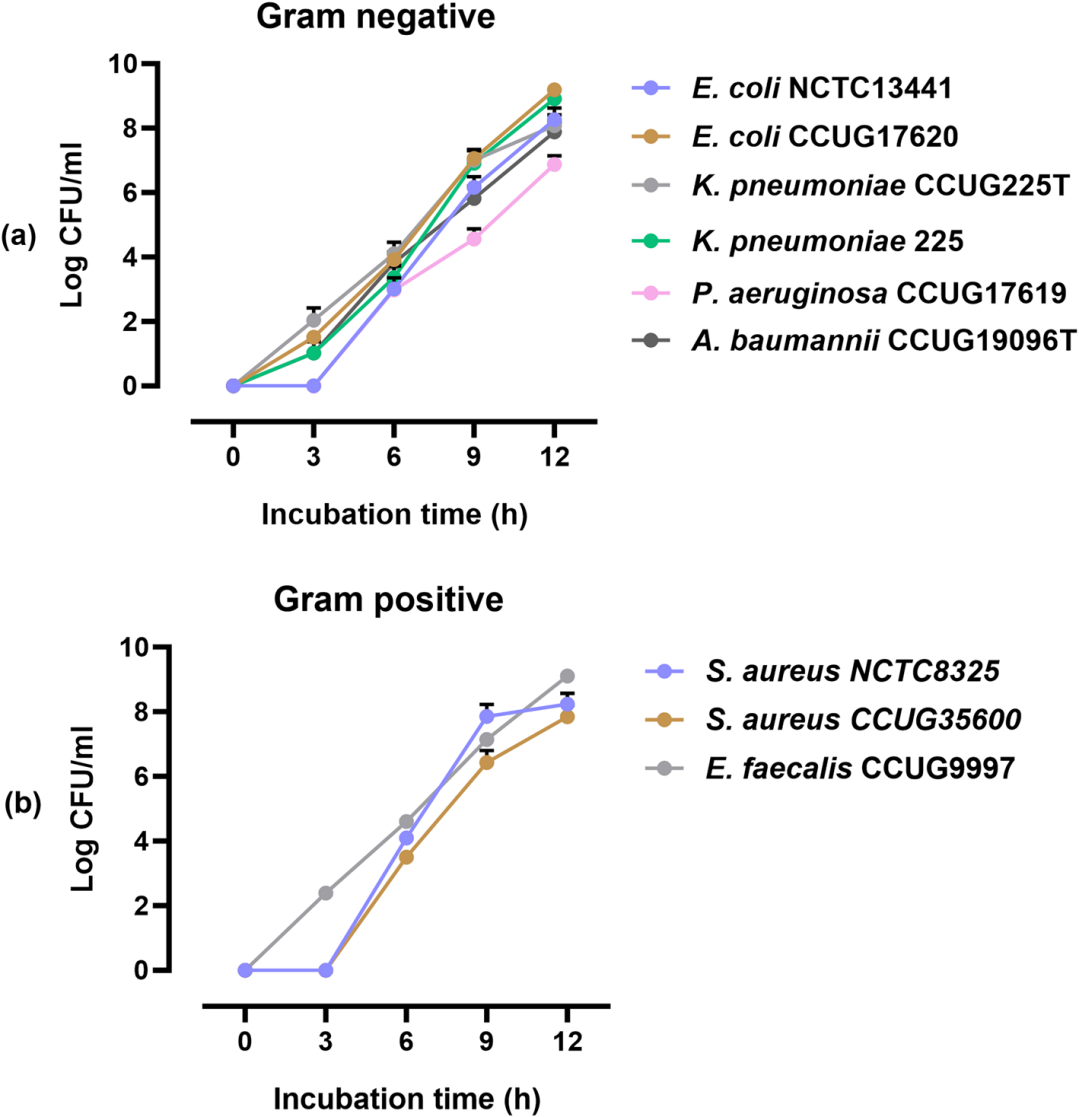
The log CFU/mL of (**a**) gram-negative and (**b**) gram-positive bacterial strains following 3, 6, 9, and 12 h of incubation. The log phase of the bacteria starts after 3 h of incubation, and the growth curve increases sharply up to 12 h.

After 9 h of incubation, the CFU/mL reached 10^3^–10^7^ for all the cultures. *E. coli* CCUG17620, *E. faecalis* CCUG9997, and *K. pneumoniae* 225 showed higher CFU/mL (10^9^) at 12 h of incubation than the other cultures. In these experiments, the bacteria continued to grow for 12 h, after which the growth was no longer measured (Fig. 1 and Table 1).

### 240 min (4 h) of incubation is considered time 0 (t_0_)

To replicate the clinical relevance, the baseline timepoint (t_0_) was determined at which the cultures reached a concentration (CFU/mL) regarded relevant for sepsis. The determination of t_0_ was crucial for aligning our experimental setup with real-world clinical scenarios. To determine t_0_, individual growth experiments were performed to monitor bacterial proliferation every 30 min after inoculation (Supplementary Table S1). Based on the results from these experiments, 240 min was established as the reference t_0_, representing the duration required for the cultures to reach the clinically significant range of CFU/mL. *E. faecalis* grew fastest, reaching 1550 CFU/mL after 240 min, while *P. aeruginosa* was the slowest growing of all the tested isolates, reaching 6 CFU/mL at 240 min (Table 1 and Supplementary Table S1). Consequently, 240 min was adopted as the uniform t_0_ for all the bacterial isolates used in this study. This standardized t_0_ value was used as the reference point for subsequent data analysis and interpretation.

### 10^2^–10^4^ CFU/mL obtained after 2 h of incubation was sufficient for pathogen detection using nanopore sequencing

The first nanopore sequencing file (comprised of 4000 reads), which became available after 10–63 min of sequencing, was enough for detecting the target bacteria at all time points and for all cultures. Information regarding the identification of bacterial strains at each time point, the reference ARGs, and the number of bacterial and human reads in each sample is provided in Supplementary Table S2.

**Figure 2:**
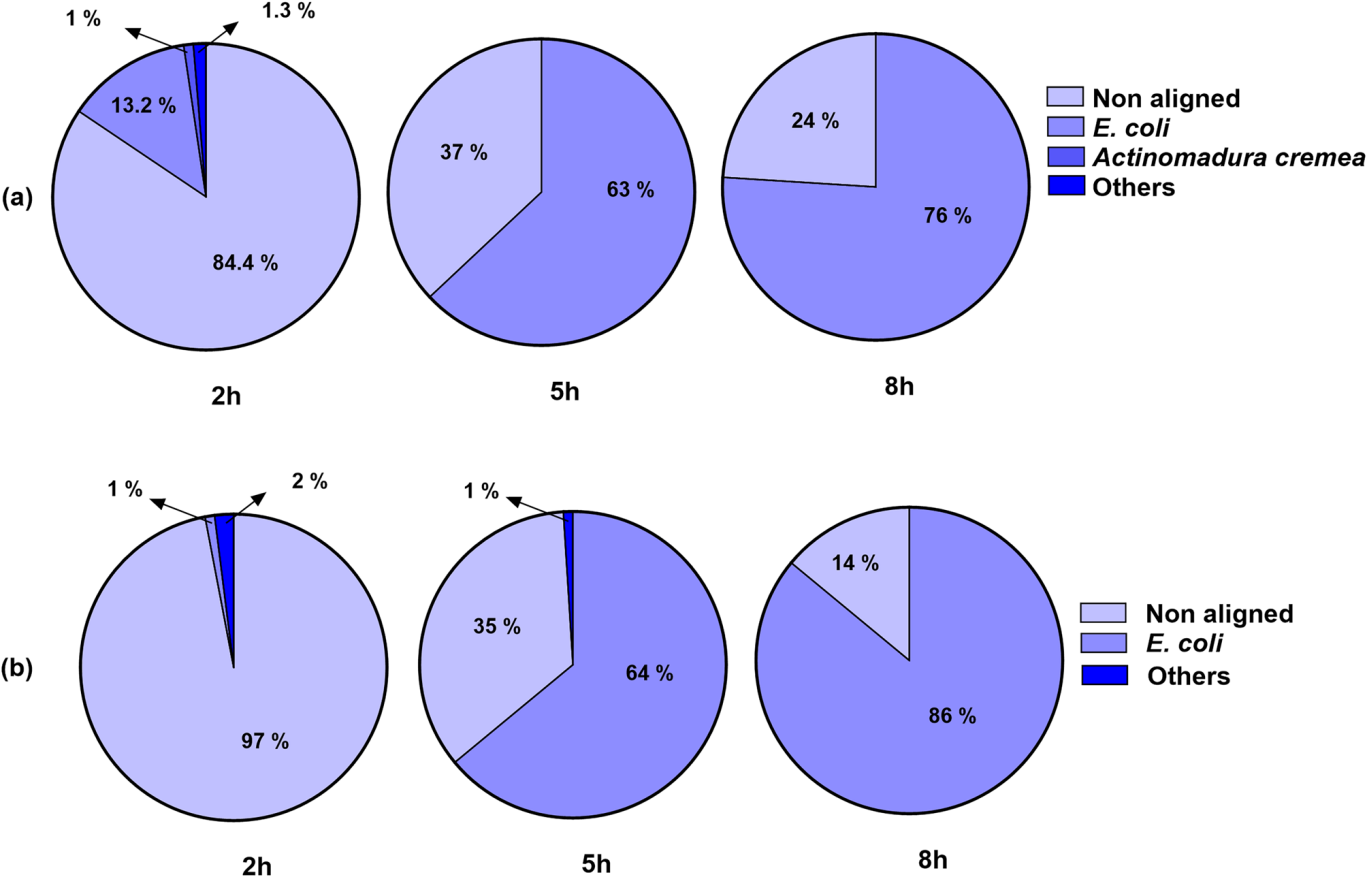
Relative distribution of sequencing reads generated by nanopore sequencing from blood cultures. The results are based on the BLAST search of the raw nanopore reads with the NCBI prokaryotic RefSeq dataset (**a**) *E. coli* CCUG17620 and (**b**) *E. coli* NCTC13441. Reads mapping to non-targeted prokaryotes are called "Others". Non aligned = human reads.

The nanopore sequencing analysis revealed the detection of all bacteria when the growth was 1.8 × 10^2^–4 × 10^4^ CFU/mL, obtained after the blood cultures were incubated for 2 h in a conventional incubator (Fig. 2; Supplementary Fig. S1; Supplementary Table S2). The *E. coli* CCUG17620, reaching 6.6 × 10^3^ CFU/mL after 2 h of incubation, showed 13% sequencing reads aligning with the targeted bacteria, which was comparatively higher than the 2-h incubated samples of the other bacteria tested. Only 1.3% of reads in this sample were associated with other prokaryotic organisms. The initial BLAST search with the RefProk database misclassified 1% of the reads as *Actinomadura cremea*. However, a BLAST search against the human genome revealed that these reads were derived from human mitochondrial DNA, which shows errors in current public database annotations.

The number of sequence reads mapping to the target bacteria increased with extended culture incubation (Fig. 2; Supplementary Fig. S1). After 5 h of incubation, *E. coli* CCUG17620 (5.7 × 10^6^ CFU/mL) and *E. coli* NCTC13441 (2.6 × 10^6^) exhibited 63% and 64% target bacterial reads, respectively. Similarly, after 8 h of incubation, 76% of sequencing reads in *E. coli* CCUG17620 (2.1 × 10^9^ CFU/mL) and 86% in *E. coli* NCTC13441 (3.5 × 10^8^ CFU/mL) blood cultures were flagged as targeted bacterial reads, respectively (Fig. 2).

The 2 h incubated sample of *S. aureus* NCTC8325, having 1.2 × 10^4^ CFU/mL, predominantly contained sequencing reads from the human genome. However, identification of the bacterium was still possible. In contrast, the 5 and 8 h incubated samples from *S. aureus* NCTC8325, having a CFU/mL of 5 × 10^6^ and 3.1 × 10^7^, exhibited 8 and 10% sequencing data specific to the target bacteria, respectively (Supplementary Fig. S1). Furthermore, the sequence data revealed that samples inoculated with *S. aureus* CCUG35600 generated the lowest bacterial reads compared to all the other samples (Supplementary Fig. S1).

The lowest CFU/mL observed to be sufficient for identifying the bacteria was 1.8 × 10^2^, recorded for *P. aeruginosa* after 2 h of incubation. Similarly, all the other bacterial pathogens were identified at around 10^3^ CFU/mL except *S. aureus* NCTC8325 and *E. faecalis,* which were detected at 10^4^ CFU/mL. These CFU/mL, which proved sufficient for detecting pathogens using nanopore sequencing, were achieved when the cultures were incubated for 2 h after reaching sepsis-relevant growth concentrations. Detailed information regarding the distribution of reads assigned to human and the targeted bacterial genome for *S. aureus*, *K. pneumoniae*, *P. aeruginosa, A. baumannii,* and *E. faecalis* can be found in Supplementary Fig. S1 and S2.

### The combination of MolYsis™ Complete5 and BiOstic bacteremia DNA kits performed better for host depletion

**Figure 3:**
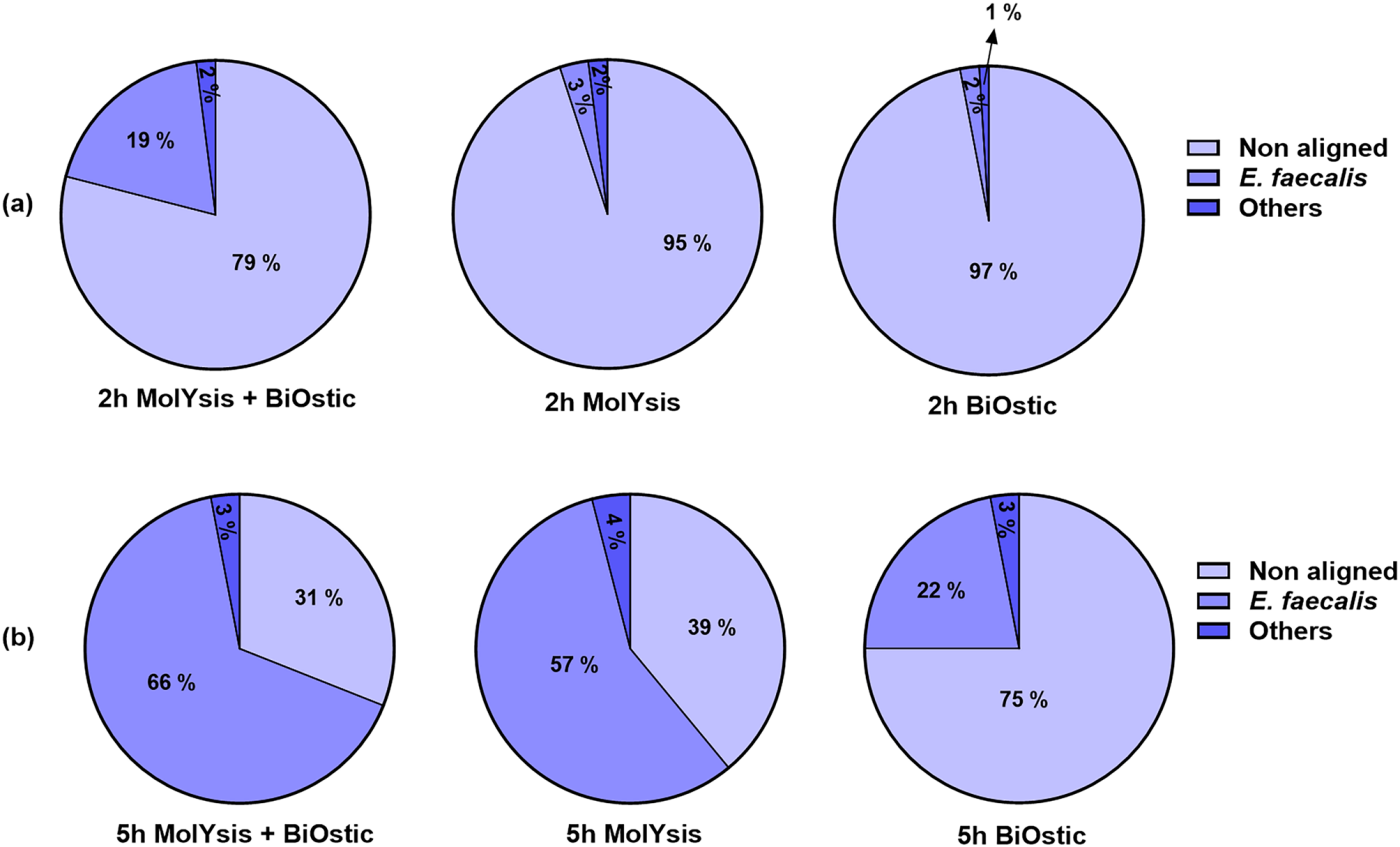
Taxonomic classification of *E. faecalis* blood culture using MolYsis™ Complete5**,** BiOstic bacteremia DNA kit and a combination of both (**a**) 2 h incubation (**b**) 5 h incubation.

Two DNA extraction kits were tested for their performance in selectively extracting bacterial DNA depleted of human DNA contaminations, and then a combination of these kits was also tested. DNA extraction from three bacterial species, *A. baumannii*, *P. aeruginosa,* and *E. faecalis,* was done using MolYsis™ Complete5 and BiOstic bacteremia DNA kit and a combination of both kits. In the 2 h incubated sample of *E. faecalis,* the MolYsis™ Complete5 and BiOstic kit alone yielded 95% and 97% human reads, respectively (Fig. 3). However, when the kits were used together, the human reads were reduced to 79%. Also, the bacterial reads increased from 2 and 3% to 19% when the kits were used in combination. Similarly, in the 5 h incubated sample of *E. faecalis,* when the kits were used in combination, the human reads were reduced from 39 and 75% to 31%, while the bacterial reads were enriched from 22 and 57% to 66% (Fig. 3).

In the 2 h incubated samples, the BiOstic kit yielded 85% and 95% human reads for *A. baumannii* and *P. aeruginosa,* respectively. However, when the BiOstic kit was used in combination with MolYsis™ Complete5, the human reads decreased from 85 to 62% for *A. baumannii* and 95% to 90% for *P. aeruginosa*. In contrast, the bacterial reads increased from 15 to 38% for *A. baumannii* and 4% to 9% for *P. aeruginosa* (Supplementary Fig. S2). Similarly, the 5 h incubated samples of *A. baumannii* and *P. aeruginosa* exhibited 71% and 96% human reads when only the BiOstic kit was used for DNA extraction. However, when the MolYsis™ Complete5 and BiOstic were used in combination, the human reads decreased from 71 to 53% in *A. baumannii* and 96% to 87% in *P. aeruginosa*. The bacterial reads increased from 29 to 47% in *A. baumannii* and 3% to 12% in *P. aeruginosa* when the MolYsis™ Complete5 and BiOstic kits were used together rather than BiOstic alone (Supplementary Fig. S2).

These results showed that DNA extraction using a combination of MolYsis™ Complete5 and BiOstic kit significantly reduces human DNA contamination while enriching the bacterial DNA from blood cultures. An initial PCR verification was also performed before DNA sequencing to confirm bacterial and human DNA (Supplementary Table S3). PCR results confirmed the nanopore sequencing results for host depletion when the MolYsis™ Complete5 and BiOstic bacteremia DNA kit were used together for DNA extraction (Supplementary Text; Supplementary Fig. S3).

### 10^3^–10^7^ CFU/mL of bacterial concentration obtained after 5 h of incubation was enough for the detection of ARGs

Clinically relevant ARGs were detected as the cultures reached 9 × 10^3^–1.1 × 10^7^ CFU/mL, achieved after 5 h of incubation when the cultures attained a sepsis-relevant concentration. Approximately 1 h of nanopore sequencing and 5 h of incubation was enough to detect all the genes except *mec*A, which was only identified when the culture was incubated for 8 h (Supplementary Table S2). A BLAST search of the sequencing data against the ABRicate database revealed that the early detection of specific genes, including *bla*EC-5, *fos*B, and *bla*SHV-164, depended on the incubation time of the cultures. Samples with longer incubation times required comparatively less sequencing time and fewer reads to detect ARGs because the DNA extracted from samples with increased incubation time contains more bacterial DNA due to higher bacterial growth.

#### *E. coli* CCUG17620

**Figure 4:**
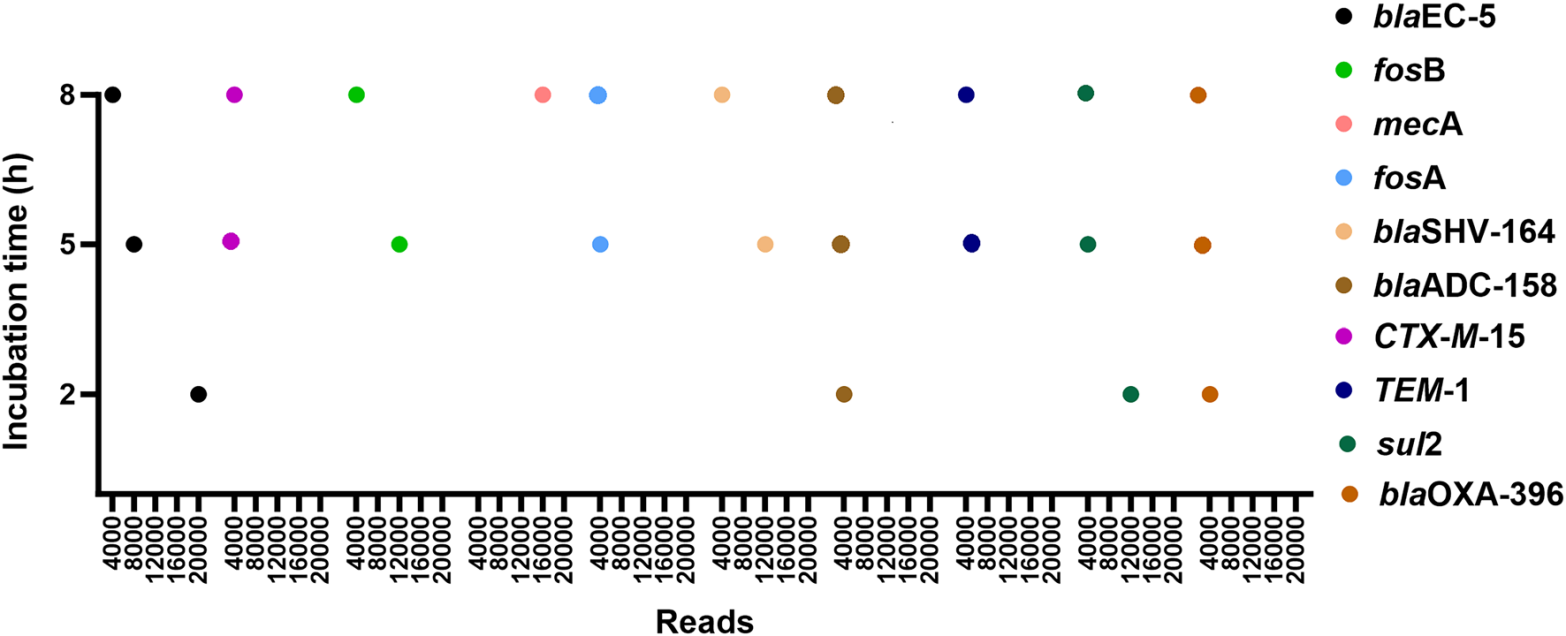
The number of nanopore reads required to detect ARGs following up to 8 h of incubation of the blood cultures. The incubation time is based on t_0._

The reference ARG *bla*EC-5 in *E. coli* CCUG17620, incubated for 2 h until the culture reached 6.6 × 10^3^ CFU/mL, required 20,000 nanopore reads (available after 60 min of sequencing) for identification. However, the presence of this gene was confirmed in the first 8000 and 4000 nanopore reads available after 20 and 10 min of sequencing in samples incubated for 5 h (5.7 × 10^6^ CFU/mL) and 8 h (2.1 × 10^9^ CFU/mL), respectively. The shortest time required to identify *bla*EC-5 was only 300 min (Fig. 4; Supplementary Table S2).

#### *E. coli* NCTC13441

The reference ARGs *CTX*-*M*-15 and *TEM*-1 were detected in the initial 4000 sequence reads (available after 25–35 min of nanopore sequencing) for culture samples incubated for 5 and 8 h, reaching 2.6 × 10^6^ and 3.5 × 10^8^ CFU/mL, respectively. The overall time required for detecting these ARGs was 455 min at 5 h and 625 min at 8 h of incubation (Fig. 4; Supplementary Table S2).

#### *S. aureus* NCTC8325

The *fos*B gene was detected after 43 min of sequencing within the first 4000 reads when the culture reached 3.1 × 10^7^ CFU/mL, achieved after 8 h of incubation. The complete process for detecting the ARG after 8 h of incubation was 643 min. Furthermore, the same *fos*B gene was also detected in the sample incubated for 5 h (5 × 10^6^ CFU/mL), requiring 95 min of sequencing and 12,000 reads. The overall time for ARG detection following 5 h of incubation and nanopore sequencing was approximately 515 min (Fig. 4; Supplementary Table S2).

#### *S. aureus* CCUG35600

Detection of the *mec*A gene in *S. aureus* CCUG35600 required 8 h of incubation (4.9 × 10^6^ CFU/mL) and 16,000 nanopore reads. The sequencing data containing information sufficient to detect the *mec*A gene took the longest time, requiring 3 h to complete. Consequently, the overall detection time for the *mec*A was 780 min (Fig. 4; Supplementary Table S2).

#### *K. pneumoniae* CCUG225T

The reference ARG *fos*A in *K. pneumoniae* CCUG225T was detected within 45 min (4000 reads) of sequencing in the sample incubated for 5 h (2 × 10^6^ CFU/mL). However, the same gene was detected within the first 4000 sequencing reads in the sample incubated for 8 h, where the CFU/mL was 1.9 × 10^7^, albeit with a detection time of 129 min. The total detection times were 465 and 729 min for samples with 5 and 8 h of incubation, respectively (Fig. 4; Supplementary Table S2).

#### *K. pneumoniae* 225

The detection of *blaSHV*-187 in *K. pneumoniae* 225 culture incubated for 5 h (1.1 × 10^7^ CFU/mL) required 62 min (first 8000 reads) of nanopore sequencing. When the culture was incubated for 8 h, the CFU/mL was 1 × 10^9^, and the detection of this gene was notably faster, taking just 24 min of sequencing. The overall detection times were 482 min for the 5 h incubated sample and 624 min for the sample with 5 h of incubation (Supplementary Table S2).

#### *A. baumannii* CCUG19096T

The reference ARG *bla*ADC-158 in *A. baumannii* was detected after 35, 27, and 22 min of nanopore sequencing in cultures incubated for 2, 5, and 8 h and having 1.6 × 10^3^, 1.6 × 10^4^ and 2.4 × 10^7^ CFU/mL, respectively. The *bla*ADC-158 gene was detected within the first 4000 nanopore reads in all samples (Fig. 4). The overall detection times were 275 min for the 2 h incubated sample and 447 and 622 min for samples with 5 and 8 h of incubation, respectively (Supplementary Table S2). The detection of the *sul*2 gene required 12,000 reads for 2 h of incubation and 4000 reads for samples with 5 and 8 h of incubation, respectively.

#### *P. aeruginosa* CCUG17619

The reference ARG *bla*OXA-396 in *P. aeruginosa* was detected in the first 4000 nanopore reads for all samples, taking 27–39 min of sequencing. The overall detection times for this gene were 279, 452, and 627 min in samples incubated for 2, 5, and 8 h, having 1.8 × 10^2^, 9 × 10^3^, and 3 × 10^6^ CFU/mL, respectively (Fig. 4; Supplementary Table S2).

#### *E. faecalis* CCUG9997

Samples were sequenced following 2 h and 5 h of incubation. The reference ARG *tet*M was detected when the CFU/mL was 10^7^ achieved after 5 h of incubation. The detection of this gene required only 4000 reads and 15 min of nanopore sequencing. The overall TAT was 440 min (Supplementary Table S2).

Other ARGs that were detected in blood culture samples followed by 2 h of incubation were *aac*(6')-Ib-cr11, *tet(*A), *mph*(A), *aac*(6')-Ib-D181Y, and *tet*(38) (Supplementary Fig. S4). Furthermore, all the reference ARGs detected following 8 h of incubation of the blood cultures were also seen at 5 h. Therefore, these results indicate that using nanopore sequencing, up to 5 h of incubation of the blood cultures where the bacterial growth was 10^3^–10^7^ CFU/mL was enough to see all expected ARGs (except the *mec*A gene in *S. aureus* CCUG35600).

The sequence reads mapping with ARGs were further BLAST searched against the plasmid database^30^. Sequencing reads carrying the β-lactamases genes (*bla*EC-5, *CTX*-*M-*15, *bla*SHV-164) were also mapped to plasmids. Meanwhile, the sequence reads hosting the *mec*A gene was not mapped with any plasmid and, therefore, flagged as chromosomal origin as previously reported^28^.

### 90% of the bacterial genome was sequenced in < 10 h of nanopore sequencing

As the time required to generate the first 4,000 sequence reads varies, we decided to present the genome coverage of the target bacteria with the number of sequences rather than the exact timeline.

#### *E. coli* CCUG17620

**Figure 5:**
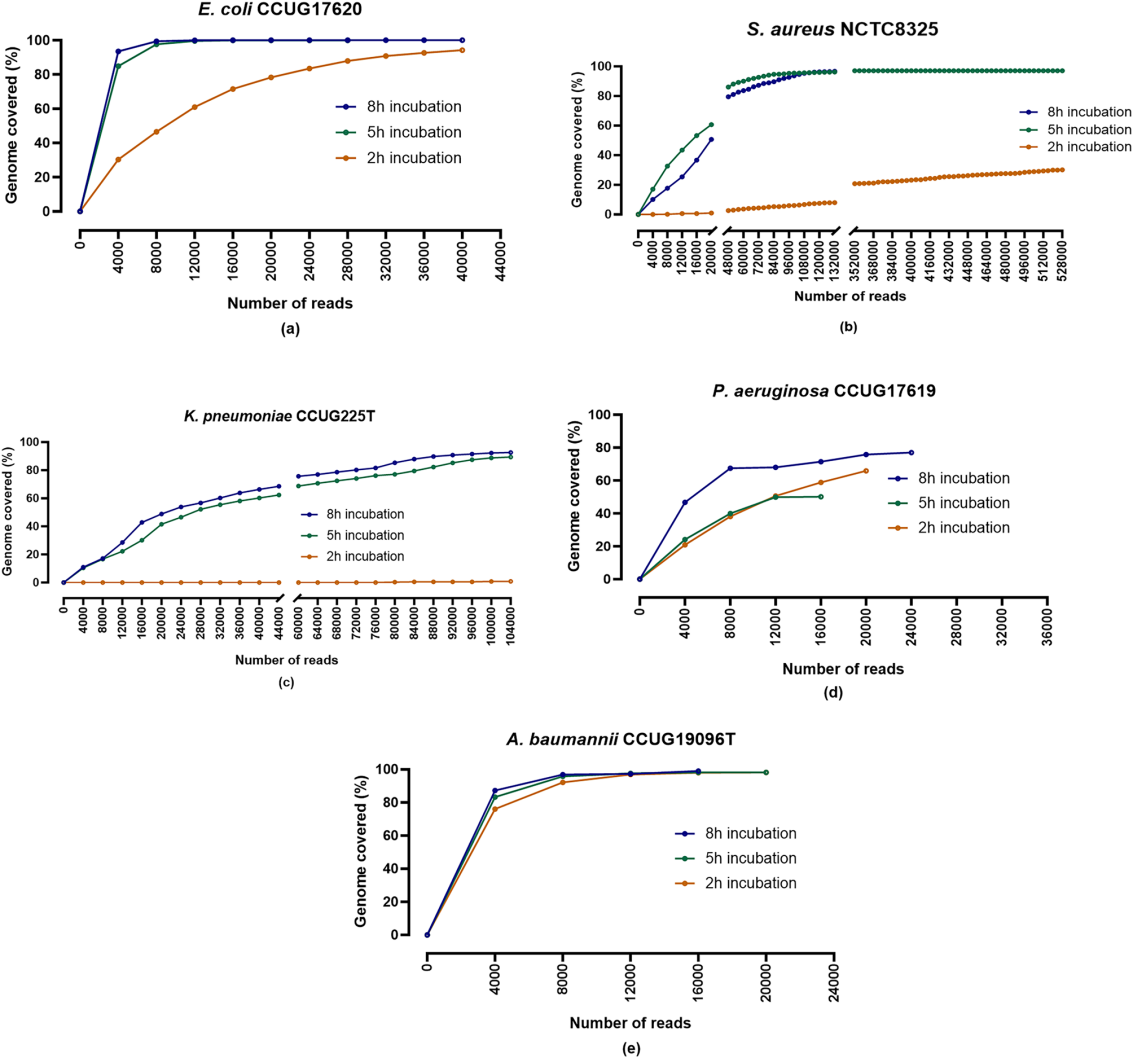
Genome coverage of the target bacterial species based on the number of nanopore sequencing reads after different incubation times. (**a**) *E. coli* CCUG17620 (**b**) *S. aureus* NCTC8325 (**c**) *K. pneumoniae* CCUG225T (**d**) *P. aeruginosa* CCUG17619 (**e**) *A. baumannii* CCUG19096T*.*

In the 2 h incubated sample containing 13% of bacterial DNA, more than 30% of the genome was sequenced within the first 4000 reads. However, 168,000 reads were required to achieve 100% genome coverage. In the 5 h incubated sample (60% bacterial DNA), full genome coverage was obtained in 32,000 reads. Similarly, 100% genome coverage was obtained within 16,000 reads in the sample incubated for 8 h. Notably, the first 4000 nanopore reads in the 5 and 8 h incubated samples were enough to sequence 85% and 93% of the genome, respectively (Fig. 5a).

#### *E. coli* NCTC13441

The maximum genome coverage of the *E. coli* NCTC13441 sample incubated for 2 and 5 h was 31% and 99% (32,000 reads), respectively. Sequence reads covered 99% of the *E. coli* NCTC13441 genome after 32,000 reads when the sample was incubated for 5 h. On the other hand, 99% genome coverage was observed after only 12,000 reads in the sample incubated for 8 h (Supplementary Fig. S5).

#### *S. aureus* NCTC8325

Genome coverage of *S. aureus* NCTC8325 at the end of the sequencing run reached 30% in the sample with 2 h of incubation. However, a genome coverage of 97% was observed when the samples were incubated for 5 and 8 h (Fig. 5b).

#### *S. aureus* CCUG35600

In the case of the MRSA strain *S. aureus* CCUG35600, 8, 16, and 58% of the genome was sequenced after 2, 5, and 8 h of incubation of the cultures, respectively. Although the *S. aureus* CCUG35600 genome was not 100% sequenced, the *mec*A resistance gene was still detected. The data showed that 16,000 reads were required for *mec*A gene identification, corresponding to 10% of the *S. aureus* CCUG35600 genome (Supplementary Fig. S5).

#### *K. pneumoniae* CCUG225T

The maximum genome coverage obtained was 1% with 2 h and 89% with 5 h of incubation (104,000 reads). Furthermore, extending the incubation for three additional hours resulted in 93% genome coverage for *K. pneumoniae* CCUG225T (Fig. 5c).

#### *K. pneumoniae* 225

The genome coverage of *K. pneumoniae* 225 reached 17% in the sample incubated for 2 h. In comparison, it required only 4000 reads to cover 41% of the genome and 56,000 reads to achieve 99% genome coverage in the sample incubated for 5 h. Similarly, in the sample incubated for 8 h, only 20,000 reads were required to sequence 90% of the genome (Supplementary Fig. S5).

#### *P. aeruginosa* CCUG17619

Notably, in *P. aeruginosa* CCUG17619, the genome coverage sequenced after 2 h of incubation was 66%, more than the sample incubated for 5 h (50%). The possible reason for this may be the exhaustion of the MinION flow cell, as these samples were run on a flow cell that was previously used and then washed (Fig. 5d).

#### *A. baumannii* CCUG19096T

The maximum genome coverage achieved by *A. baumannii* was 99% at 8 h of incubation, requiring 16,000 sequencing reads. Similarly, 20,000 nanopore reads were required for the samples incubated for 2 and 5 h to achieve 98% genome coverage (Fig. 5e).

#### *E. faecalis* CCUG9997

The first 4000 nanopore reads provided a genome coverage of 25% and 38% after 2 and 5 h of incubation, respectively. The total genome sequenced was 49%, requiring 12,000 nanopore reads at 2 h of incubation. However, 99% genome coverage was obtained after 5 h of incubation of the sample requiring 56,000 sequencing reads (Supplementary Fig. S5).

### Flongle sequencing complements MinION for pathogen identification

The 2 h incubated sample of *E. coli* CCUG17620 and 5 h sample of *K. pneumoniae* CCUG225T were also sequenced using a Flongle flow cell. The MinION sequencing for these bacterial strains identified the pathogen and its ARGs (Fig. 2a; Supplementary Fig. S1c). The Flongle data were enough to detect both pathogens, but identifying all the relevant reference ARGs was possible in only one sample (*K. pneumoniae* CCUG225T). The *E. coli* sample*,* when sequenced using Flongle, showed 3% of the reads assigned to the *E. coli* and 1.6% to *K. pneumoniae*. The most probable reason for this misclassification may be the high similarity of *K. pneumoniae* and *E. coli* genomes. For *K. pneumoniae,* 4.2% of reads were assigned to *K. pneumoniae*, and 1.3% were misclassified as other prokaryotes. All the relevant ARGs were detected in *K. pneumoniae*. However*,* using Flongle data, the exact variant of the reference ARG was not detected in *E. coli*.

### Four to Nine hours of turn-around-time (TAT) from sample collection to pathogen identification and ARG detection

#### Two hours of incubation

Correct bacterial identification was possible between 20–40 min of sequencing. However, ARGs were only identified in three bacteria, including *E. coli* CCUG17620, *P. aeruginosa,* and *A. baumannii*. The ARGs detection time for these bacteria was between 35 and 60 min of sequencing run. Therefore, the TAT, including bacterial incubation, DNA extraction, library preparation, nanopore sequencing, and real-time data analysis, was 260–280 min for bacterial ID and 275–300 min for ARGs detection (Supplementary Table S2).

#### Five hours of incubation

The identification of bacteria was possible between 20–45 min of nanopore sequencing. All the reference ARGs (except *mec*A) were identified between 20 and 95 min of the sequencing run. The overall TAT for the detection of bacteria was between 440 and 465 min, and for the detection of ARGs, it was between 440 and 515 min (Supplementary Table S2).

#### Eight hours of incubation

10–63 min of nanopore sequencing was sufficient for detecting bacteria. However, 10–180 min of nanopore sequencing were required to identify all the reference ARGs. Thus, the overall TAT for the pathogen detection was between 610 and 663 min, and for the ARGs detection, it was between 610 and 780 min (Supplementary Table S2).

**Figure 6:**
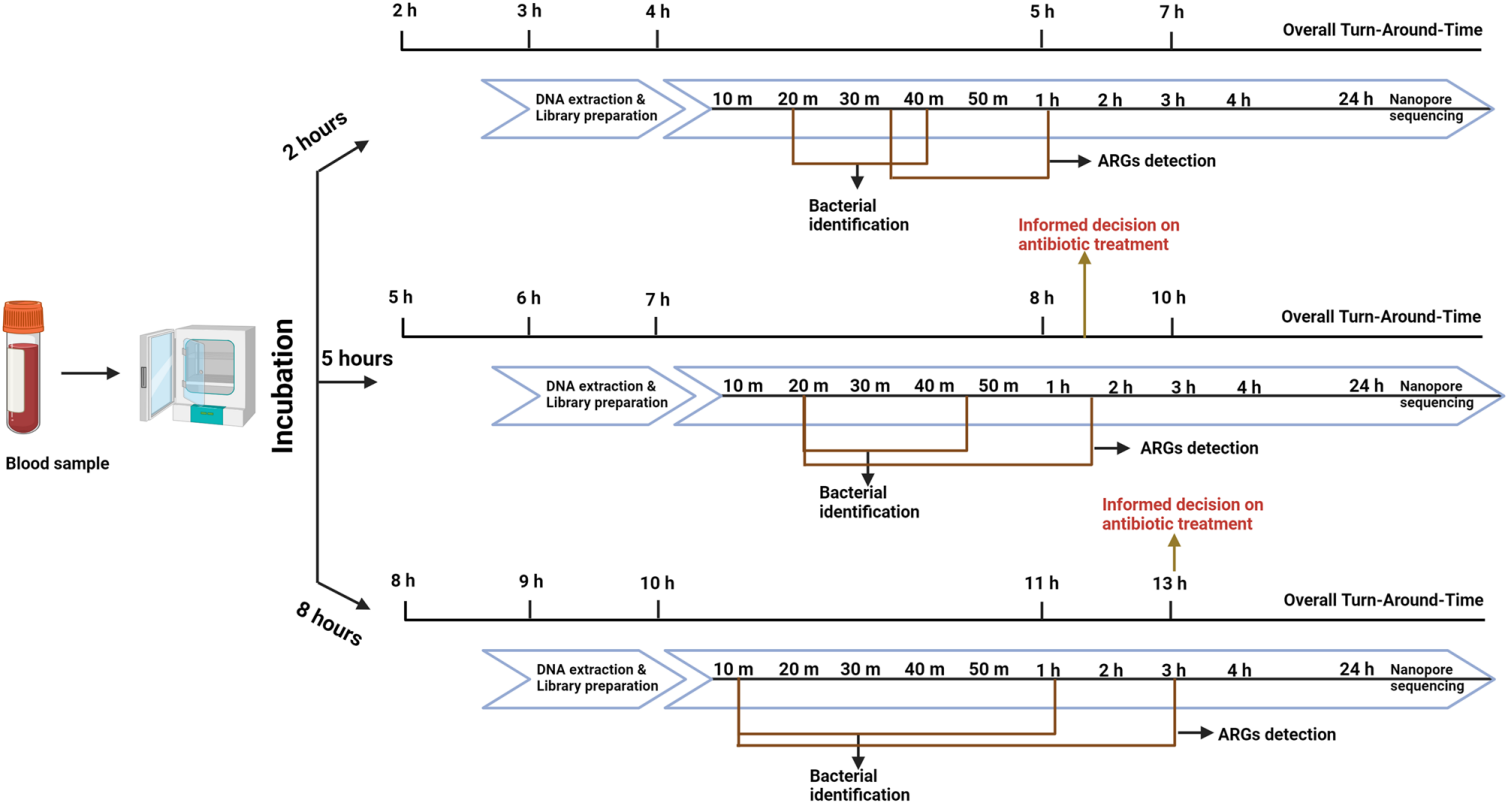
Timeline for the detection of pathogens and ARGs from blood culture samples. The timeframe for identifying pathogens and ARGs is calculated using t_0_.

In summary, all the bacterial pathogens were identified by growing the cultures to a density of 10^2^–10^4^ CFU/mL, requiring 2 h of incubation. However, identifying all the relevant ARGs was possible with CFUs ranging between 10^3^ and 10^7^, observed when the cultures were incubated for 5 h. Interestingly, some reference ARGs were detected from samples sequenced after 2 h of incubation. All reference ARGs (except *mec*A, identified after 8 h of incubation) were identified by sequencing the 5 h of incubated samples (Supplementary Table S2). Therefore, the shortest time (260–300 min, ca. 5 h) to detect sepsis-causing bacteria and ARGs was seen for *E. coli* CCUG17620, *A. baumannii* and *P. aeruginosa*, and the longest time (13 h) was required for the detection of *mecA* gene in *S. aureus* CCUG35600 (Fig. 6). However, the bacterial ID was possible in less than 5 h, even for the *S. aureus* CCUG35600 sample.

Figure 6 shows the timeline for the identification of bacterial pathogens and ARGs from samples with incubation times of 2, 5, and 8 h. DNA extraction from the blood cultures and library preparation for nanopore sequencing required 2 h. For the samples incubated for 2 h, 20–40 min of nanopore sequencing was enough to identify the pathogen, corresponding to a TAT of 260–280 min (ca. 4.5 h). Similarly, for the 5 h incubated samples, the TAT from the sample preparation until an informed decision on which antibiotic treatment should be prescribed was 515 min (ca. 8.5 h). From these results, it can be concluded that 10^3^–10^7^ CFU/mL of bacterial concentration in blood culture was enough to detect the pathogen ID and all the clinically relevant ARGs. However, the identification of pathogen ID was even possible at lower CFUs (≥ 10^2^ CFU/mL).

## Discussion

Rapid diagnosis of blood cultures from sepsis patients is crucial to promptly prescribing the appropriate antibiotics. This study investigated reducing the wait time for sepsis diagnosis by employing shorter blood culture times followed by nanopore sequencing. All the tested pathogens and their associated ARGs were detected from cultures having 10^3^–10^7^ CFU/mL, achieved after 5 h of incubation and 20–95 min of nanopore sequencing. Therefore, the total TAT ranged between 7 and 9 h (440–515 min) from sample collection, which included sample incubation, library preparation, sequencing, and data analysis. Interestingly, taxonomic identification of all the tested bacteria and some ARGs (*bla*EC-5, *bla*ADC-158, and *bla*OXA-396) was successful when the CFU/mL was between 1.8 × 10^2^–4 × 10^4^, obtained after 2 h of incubation and 20–60 min of nanopore sequencing. Therefore, the shortest TAT was approximately 260 min for pathogen ID and 275 min for the detection of ARG.

The results of this study showed that the limit of detection of pathogens was between 10^2^ and 10^4^ CFU/mL, obtained at 2 h of incubation after the cultures reached a sepsis-relevant growth concentration. The lowest concentration recorded to detect the pathogen was 10^2^ CFU/mL for *P. aeruginosa* culture. The slower growth of *P. aeruginosa* compared to other pathogens leads to a longer time to positivity in blood cultures and has been reported previously^31–33^. Two isolates, *S. aureus* NCTC8325, and *E. faecalis,* had a higher CFU/mL (10^4^) at 2 h of incubation, where it was detected. All the other cultures had a CFU of around 10^3^ when identified using nanopore sequencing.

However, identifying all the relevant reference ARGs was successful when the bacteria were grown between 10^3^ and 10^7^ CFU/mL, requiring 5 h of incubation after the cultures reached sepsis-relevant growth. The lowest CFU at which the ARG was detected was 10^2^_,_ which was recorded for *P. aeruginosa* after only 2 h of incubation. However, the highest growth concentration (10^7^ CFU/mL) at which the target ARGs (*bla*SHV-187 and *tet*M) were detected was observed for *K. pneumoniae* 225 and *E. faecalis* at 5 h of incubation. All the other tested pathogens had a CFU between 10^3^ and 10^6^ CFU/mL until relevant ARGs were detected.

### Two hours of incubation of the blood cultures in a standard laboratory incubator is sufficient for bacterial ID

Previous studies using nanopore sequencing from blood cultures have analyzed blood cultures grown for up to 24 h and flagged positive by the automated incubation system^26,28,34^. Clinical and Laboratory Standards Institute (CLSI) guidelines and the 2016 American Society for Microbiology Clinical Microbiology Procedures Handbook recommend a blood culture incubation period of 120 h^36,37^. Shorter durations (72–96 h) have also been reported as appropriate for automated blood culture systems^38–40^. Incubation in the automated system significantly increases the bacterial load in the blood cultures as bacteria grow. However, these automated blood culture instruments are expensive, costing several thousand US dollars, and unavailable in small-scale laboratories. Moreover, the delay caused by the transport of inoculated blood culture bottles to the central laboratory with the incubation instrument can increase the time of pathogen detection, which is not desirable for patients^41–45^. In the future, this method, where we have used standard laboratory incubators to incubate the blood cultures, can reduce the costs associated with automated blood culture incubation instruments and transport.

Loonen et al. used a similar reduced incubation approach in blood cultures inoculated with *S. aureus* and *E. coli*. They tried to identify the bacteria after 7 h of incubation using MALDI-TOF–MS. However, their findings indicated that despite using multiple MALDI-TOF approaches, it was not feasible to identify both bacterial isolates after 7 h of incubation^46^. Subsequently, they extended the incubation of the blood cultures to determine the time required for both bacterial species to yield positive results. However, accurately identifying the isolates using MALDI-TOF was possible only when the samples were flagged positive (taking 12–19.5 h) by the BacT/ALERT 3D system^46^. MALDI-TOF MS has effectively identified pathogens from positive blood culture bottles and is considered a promising approach for rapid diagnostics^47–49^. However, MALDI-TOF has several limitations, including the initial cost of the instrument (> 250,000 US$)^34^, poor identification in the case of polymicrobial infections^50^, and antibiotic resistance determinants^51–53^.

Our results show that a bacterial concentration as low as 10^2^ CFU/mL, achieved after 2 h of incubation of sepsis-relevant growth (t_0_), was sufficient for bacterial ID using nanopore sequencing. The first 4000 raw sequencing reads available within 40 min of nanopore sequencing were enough for bacterial identification with a high degree of certainty. In a similar study, Taxt et al. identified pathogens from positive blood cultures in the first sequencing output file comprising 4000 nanopore reads^28^. Sakai et al. have also reported that 30 min of nanopore sequencing coupled with the ONTs What's In My Pot (WIMP) and Antimicrobial Resistance Mapping Application **(**ARMA) workflows could provide information regarding the gram-negative bacteria for BSIs and the relevant ESβL genes^34^.

Several previous sequencing-based studies to detect pathogens from blood cultures have used higher pathogen concentrations (10^7^–10^9^ CFU/mL)^16,22,28,54^. Here, we wanted to find the limit of detection of bacterial pathogens in blood cultures. Therefore, we have sequenced samples with varying CFUs obtained when the cultures were grown for different periods. We identified all the tested bacterial pathogens from blood cultures with CFUs as low as 10^2^–10^3^, except *S. aureus* NCTC8325 and *E. faecalis*, identified at 10^4^ CFU/mL. The sensitivity of pathogen detection varied across the samples, with *E. coli* samples showing more supporting reads than the others. Even with fewer supporting reads and more human reads in the other isolates, the identification of pathogens was still successful, as reported previously^55^.

Further optimization of the DNA extraction from blood cultures to deplete the human/ host DNA at lower concentrations will enhance the sensitivity of bacterial detection. For this purpose, we used the MolYsis™ Complete5 and BiOstic bacteremia DNA kit and a combination of both kits to extract DNA from *P. aeruginosa*, *E. faecalis,* and *A. baumannii*. The results showed that the detection sensitivity was higher when both the kits were used together, with more sequencing reads mapping to the target bacteria. It has been demonstrated that when MolYsis™ Complete5 is used in combination with ox bile and nuclease, it enriches the bacterial DNA and depletes the host^56^.

### Clinically relevant ARGs were targeted for the detection

The tested clinical strains have reference assemblies available in public repositories. So, the ground truth, including the ARGs in these isolates, was known, and we were able to detect all these relevant ARGs in this study. The clinically significant ARGs targeted for detection were *bla*EC-5, *CTX*-*M*-15, *TEM*-1, *fos*B, *mec*A, and different variants of *bla*SHV. These ESβL and MRSA (Methicillin Resistant *Staphylococcus aureus*) phenotypes of *Enterobacteriaceae* and *S. aureus* are prevalent worldwide. The WHO lists them as priority pathogens for which new antibiotics are urgently required^28^. Here, we detected all these genes (except *mec*A) after only 5 h of incubation and 1 h of sequencing except *fos*B, which took around 95 min. These results align with previous studies showing the detection of ARGs in *K. pneumoniae* positive blood cultures after 1–2 h of nanopore sequencing^55,57^. The *S. aureus mec*A gene was only detected in the sample incubated for 8 h, requiring 16,000 reads from 3 h of sequencing. It is similar to what was observed for this gene in our previous study, where the detection required a longer time (around 16 h) than any other ARGs^28^. The possible reason for this extended time to detection of the *mec*A gene may be the slow growth of the *S. aureus,* having 4.9 × 10^6^ CFU/mL after 8 h of incubation and hence less bacterial DNA obtained after extraction. Also, *S. aureus* is a gram-positive bacterium, and DNA extraction might be more challenging due to the thick peptidoglycan layers surrounding the cell wall.

As described previously, sequencing-based studies have primarily relied on ARGs detection from positive blood cultures initially incubated in the blood culture incubation systems and tagged positive. Therefore, we were interested in finding the detection limit for ARGs in blood cultures. The results showed the identification of all the clinically relevant ARGs in the tested bacterial species between 10^2^ and 10^7^ CFU/mL. We detected the reference ARG (blaOXA-396) in *P. aeruginosa* when the culture was grown to a CFU as low as 10^2^, requiring 2 h of incubation. All the other ARGs were identified when the cultures reached a growth concentration of 10^3^–10^7^ CFU/mL, obtained after 5 h of incubation, except *S. aureus* CCUG35600, which required 8 h of incubation.

### Nanopore sequencing can potentially be an effective tool in the future for clinical diagnostics

Nanopore sequencing can overcome most of the limitations current blood culture diagnostic methods face. It offers several advantages, like low cost, portability, real-time sequencing and data analysis, easy library preparation protocols, and a wide variety of sequencing kits. The cost of the ONT starter pack is $1999, which includes the MinION device, sequencing kits, and two flow cells (https://store.nanoporetech.com/minion.html). In our experiments, we used the rapid barcoding and ligation sequencing kits (coupled with barcode expansion), which allowed us to run multiple samples in one sequencing run by barcoding them, thus further reducing the costs. Using this methodology, we estimate the cost per sample to be ca. $40, including the costs of culture media, DNA extraction kit, library preparation kit, and flow cell. Also, sequencing costs are gradually decreasing, and different flow cells (MinION, Flongle, and PromethION) are available per user needs.

Moreover, the Flongle flow cell device offered by ONT is cheaper and can significantly reduce the costs per sequencing run^16^. Flongle sequencing complements MinION for detecting pathogens but could not detect all the relevant ARGs. This is consistent with our previous study showing that Flongle sequencing could effectively identify pathogens and ARGs. But compared to MinION, the Flongle performed poorly in ARG variant detection and sequencing multiple samples per flow cell^16^.

In summary, this study has shown the accurate identification of the bacterial ID and detection of ARGs using nanopore sequencing by incubating blood cultures for as little as 2 h in a standard laboratory incubator. However, 5 h of incubation was required to sequence the bacterial genome completely and obtain information about all the ARGs. Bacterial identification and ARG detection were possible in 10–40 min and 10 min—3 h of sequencing, respectively. We have shown that the bacterial pathogens from blood cultures can be detected in approximately 260 min when the cultures are incubated for 2 h. For the detection of ARGs, the shortest time achieved was 275 min (Supplementary Table 5). The complete process required as little as 7 to 9 h from sample collection until the bacterial ID and AMR profile were detected. This method can be advantageous in the future for diagnosing BSIs by significantly reducing the time to detect pathogens and providing early information to clinicians for prescribing appropriate antibiotics. Such a workflow can be vastly impactful in handling and preventing the spread of AMR and could lead to potential future use in clinical microbiology. The development and further validation of this sequence-based approach can reduce the burden of broad-spectrum and empirical antibiotic therapy.

The timeline for detecting pathogens and ARGs in the current study is based on the calculated t_0,_ as explained previously. Based on our study, we recommend that the cultures be grown to the desired concentration in the blood culture medium and then used as an inoculum for a new experiment. In this way, the lag time of the bacteria (t_0_) can be diminished as bacteria are transferred to the same medium. Similarly, the time variation of the growth was different for different species and strains of the bacteria. Therefore, more bacterial strains should be tested for their growth in blood cultures. Future research should focus on using this method on actual clinical samples from the patients by incubating them in traditional laboratory incubators instead of automated blood culture systems and comparing it with the clinical routine methods used for diagnosing sepsis.

## Methods

### Ethics statement

Human blood samples were obtained from anonymous healthy donors at the Department of Biotechnology, Inland Norway University of Applied Sciences (INN). Blood samples were spiked with different bacterial strains, as mentioned below. In line with our previous work^16^, there was no intention to sequence human DNA. Therefore, QIAamp BiOstic Bacteremia and MolYsisTM Complete5 kits for bacterial DNA extraction were used. Moreover, any sequencing reads recognized as being generated from human DNA were omitted from further analysis and permanently discarded. The microbiology laboratory at INN is approved for the described experimental work. All methods were carried out in accordance with relevant guidelines and regulations of the INN University. All the participants were older than 18 years of age, and informed consent was obtained before processing the blood samples.

### Bacterial strains

In this study, the most common sepsis-causing bacteria, including *Escherichia coli*, *Staphylococcus aureus, Klebsiella pneumoniae*, *Acinetobacter baumannii*, *Enterococcus faecalis,* and *Pseudomonas aeruginosa,* have been used for their limit of detection in blood cultures (Supplementary Table S4). One antibiotic susceptible (S) and one resistant strain (R) of each *E. coli*, *S. aureus,* and *K. pneumoniae,* while one R strain of each *A. baumannii*, *E. faecalis,* and *P. aeruginosa* were used to spike the healthy and fresh human blood samples. The antibiotic-resistant isolates of *E. coli* and *K. pneumoniae* were Extended-spectrum beta-lactamase (ESβL) positive, and *S. aureus* was a methicillin-resistant strain. The *E. coli* strain CCUG17620 (S), *K. pneumoniae* CCUG225T (S), *A. baumannii* CCUG1909 (R), *E. faecalis* (R) *P. aeruginosa* CCUG17619 (R), and *S. aureus* CCUG35600 (R) were obtained from the Culture Collection University of Gothenburg (CCUG, Sweden). The *E. coli* strain NCTC13441 (R), carrying the *CTX*-*M*-15 ESβL gene, and susceptible *S. aureus* NCTC8325 were obtained from the National Collection of Type Cultures (NCTC) Public Health England. All bacterial strains were stored at − 80 °C in a glycerol stock until one day before the experiment.

### Preparation of spiking inoculum for blood cultures and CFU counts

The frozen bacterial isolates (− 80 °C in glycerol) were revived by inoculating Brain Heart Infusion (BHI) agar medium and incubated at 37 °C overnight. Three to four colonies from each of the overnight cultures were suspended in separate tubes containing 1 mL of 1X Phosphate Buffered Saline (PBS) (VWR life sciences), and bacterial suspension’s absorbance at 600 nm (A600) was measured spectrophotometrically (Thermo Electron Corporation, BioMate 3, USA) (Supplementary Table S5). To determine the number of viable cells, each bacterial suspension was further serial diluted (up to 10^–6^ in PBS), and 50 µL aliquots were plated on BHI agar plates to determine colony-forming units per mL (CFU/mL). Multiple bacterial suspensions were prepared to investigate the correlation between A600 and CFU/mL for each bacterial strain used in the study (Supplementary Table S5). To mimic the clinical sepsis levels, the number of viable cells in the blood-spiking bacterial inoculum was set to < 50 CFU/mL. A600 values were only used to estimate the number of cells/mL in the inoculum, and exact CFU counts were performed using plate counting as described previously (Supplementary Fig. S6).

### Inoculation of blood culture media

**Figure 7:**
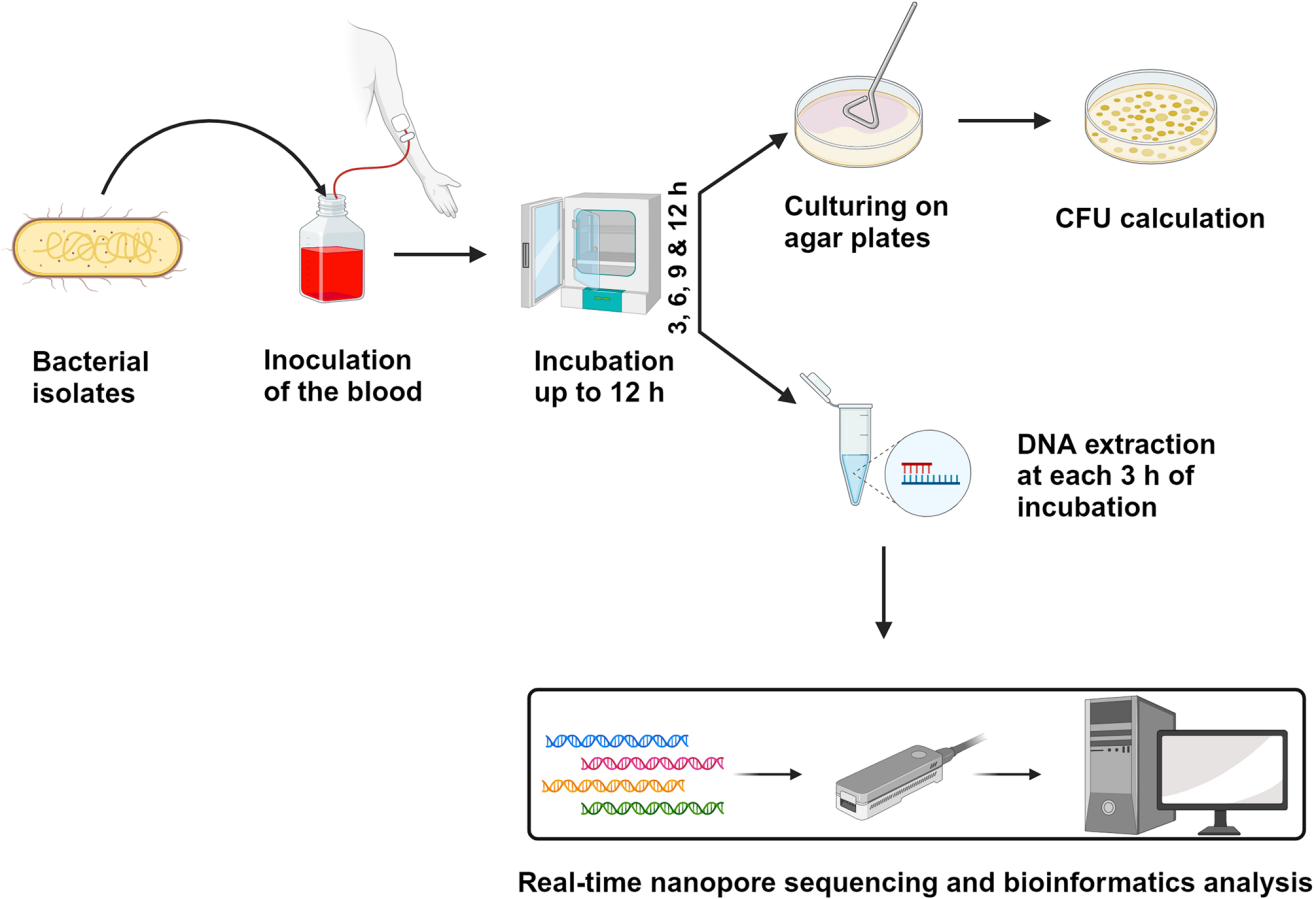
Overview of the steps involved in detecting bacteria and antibiotic resistance genes from spiked blood samples at different incubation times using nanopore sequencing (Created with BioRender.com).

5 mL blood samples from healthy volunteers were added to BD BACTEC™ Plus Aerobic medium (BD) culture bottles. 1 mL fresh bacterial inoculum, estimated to have 10–50 CFU/mL, was added to the blood culture bottles. The experiments continued only if the spiking inoculum contained < 50 CFU/mL. Initiated blood spiking experiments where the CFU/mL did not meet the < 50 CFU/mL criteria were terminated. Cultures without added bacterial inoculum were used as controls to validate the sterility of the blood. The blood cultures were incubated at 37 °C and 200 revolutions per minute (RPM) in a standard laboratory incubator. Bacterial growth was monitored for up to 12 h by drawing samples from the culture at four different time points (3, 6, 9, and 12 h) and determining the CFU/mL using plate counts on BHI agar plates. Simultaneously, 2 mL samples from each time point were stored at − 20 °C for later DNA extraction (Fig. 7).

### Time "0" (t_0_) calculation

In this study, t_0_ refers to the time the number of viable cells reaches a concentration of 50 CFU/mL in the blood culture after inoculation. Because the blood cultures were spiked with 1 mL of varying concentrations (but all below 50 CFU/mL) of cells, the final and initial concentrations of cells in the different blood cultures varied, influencing the duration of the lag phase, defined as the initial period in the life of a bacterial population when cells are adjusting to a new environment. Therefore, it is essential to calculate the t_0_ for all species used. The t_0_ calculation was done to mimic clinical sepsis, where the bacterial growth is already in the exponential phase at the time of sample collection from septic patients, and the CFU/mL is usually less than 100. So, to make these experiments relevant to the clinical scenario in the case of sepsis, the incubation time in this study was calculated by subtracting the t_0_.

### DNA extraction and nanopore sequencing

DNA extraction from the blood culture samples, which were sampled from the BACTEC bottles after each 3 h of incubation, was performed for *E. coli*, *K. pneumoniae,* and *S. aureus* using QIAamp BiOstic Bacteremia Kit (Qiagen, Germany) as described previously^28^. For extraction of DNA from *A. baumannii*, *E. faecalis,* and *P. aeruginosa*, in addition to the BiOstic Bacteremia Kit, we also tested the MolYsis™ Complete5 (Molzym GmbH & Co. Bremen, Germany) and a combination of both. The concentration and purity of the DNA were checked using a Qubit 2.0 fluorometer (ThermoFisher Scientific) and Nanodrop spectrophotometer (ND-1000). The Agencourt AMPure XP system (Beckman Coulter, USA) was an optional step to purify and enrich the DNA. The library preparation for nanopore sequencing was performed using the Rapid Barcoding Sequencing Kit (SQK-RBK004) and Ligation Sequencing Kit (SQK-LSK109) coupled with Native Barcoding Expansion 1–12 (PCR-free-EXP-NBD104) (Oxford Nanopore, UK) according to the manufacturer's protocol. Sequencing was performed on MinION (R9.4.1 FLO-MIN 106) and Flongle flow cells (R9.4.1 FLO-FLG001). Raw sequencing data were collected using ONT MinKNOW GUI software (version 5.0.0). The real-time base calling using FAST mode was also performed using ONT MinKNOW GUI software. Later, raw fast5 data were basecalled in the high accuracy mode, demultiplexed, and trimmed for the barcodes/adapters using Guppy stand-alone software (version 6, Oxford Nanopore).

### Bioinformatic analysis

#### Pathogen and antibiotic resistance gene (ARG) Identification

The MinKNOW software continuously generates sequencing data, providing 4000 sequences per file using the default settings. The output files provided were analyzed using our in-house developed bioinformatics pipeline for taxonomic identification (using Kraken2 and BLAST) and ARG identification. In addition, reads were BLAST searched against the NCBI RefProk database for taxonomic classification and mapped against the ABRicate database for ARG detection. The reference assemblies for the strains used in this study can be found in the NCBI database by using accession numbers (GCF_900448475.1, GCF_017357505.1, GCF_000240185.1, GCF_000013425.1, GCF_009035845.1, GCF_024507955.1) and also in our previously published studies^16,28,58^. Therefore, we knew the ground truth about the antibiotic resistance profiles for each bacterial sample. Reads recognized as human were omitted from further analysis and discarded by the pipeline.

#### Genome coverage analysis

Assembly files for each spiked isolate were downloaded and indexed using the *makeblastdb* option in BLAST. Later, output files generated from the nanopore sequencing for each isolate were BLAST searched against their corresponding and indexed reference genome to find the genome coverage of the generated data. Genome coverage was calculated as the proportion of the reads mapped against the reference genome. The genome coverage analysis was performed in MATLAB R2022a.

Unless otherwise stated, all graphs and figures in this study are created using GraphPad Prism (10.0.2) and BioRender.com.

### Supplementary Information

Supplementary Information.

## Data Availability

The datasets presented in this study can be found in online repositories. The names of the repository/repositories and accession number(s) can be found below: https://www.ebi.ac.uk/ena, PRJEB70780.
