# Supplementary material for: Short turnaround time of seven to nine hours from sample collection until informed decision for sepsis treatment using nanopore sequencing
Source: Sci Rep. 2024 Mar 19;14:6534. doi: 10.1038/s41598-024-55635-z (PMC10951244; doi:10.1038/s41598-024-55635-z)
Supplement: Supplementary file 1 — Supplementary Information. [file 41598_2024_55635_MOESM1_ESM.pdf]

Short turnaround time of seven to nine hours from sample collection until informed decision for sepsis treatment using nanopore sequencing

Jawad Ali[1], Wenche Johansen[1], and Rafi Ahmad[1,2*]

[1]Department of Biotechnology, Inland Norway University of Applied Sciences, Holsetgata 22, 2317, Hamar, Norway.

[2]Institute of Clinical Medicine, Faculty of Health Sciences, UiT - The Arctic University of Norway, Hansine Hansens veg 18, 9019, Tromsø, Norway

Email[*]: rafi.ahmad@inn.no

Supplementary Text

As an initial verification of human DNA depletion, PCR was performed using *A. baumannii* and *P. aeruginosa* specific primers amplifying a 300 bp fragment of gene *gyr*B and 325 bp fragment of *phz*A2 gene, along with a primer set amplifying a 100 bp fragment of the human β-actin gene to confirm the presence of bacterial and human DNA in the extracted samples (Supplementary Table S3). The visualization of the PCR product on agarose gel showed fragments of the expected sizes for all DNA samples. The *gyr*B gene fragment for *A. baumannii* and *phz*A2 gene fragment for *P. aeruginosa* showed sharp bands compared to the human β-actin gene fragment in all samples. However, samples extracted with MolYsis^{TM} Complete5 and a combination of both the kits (in *A. baumannii)* showed weaker or no bands on the agarose gel for the human β-actin gene (Supplementary Fig. S3). These PCR results confirmed the nanopore sequencing results, particularly in *A. baumannii* samples, in which the depletion of the host DNA and enrichment of the bacterial DNA were observed when both kits were used together.

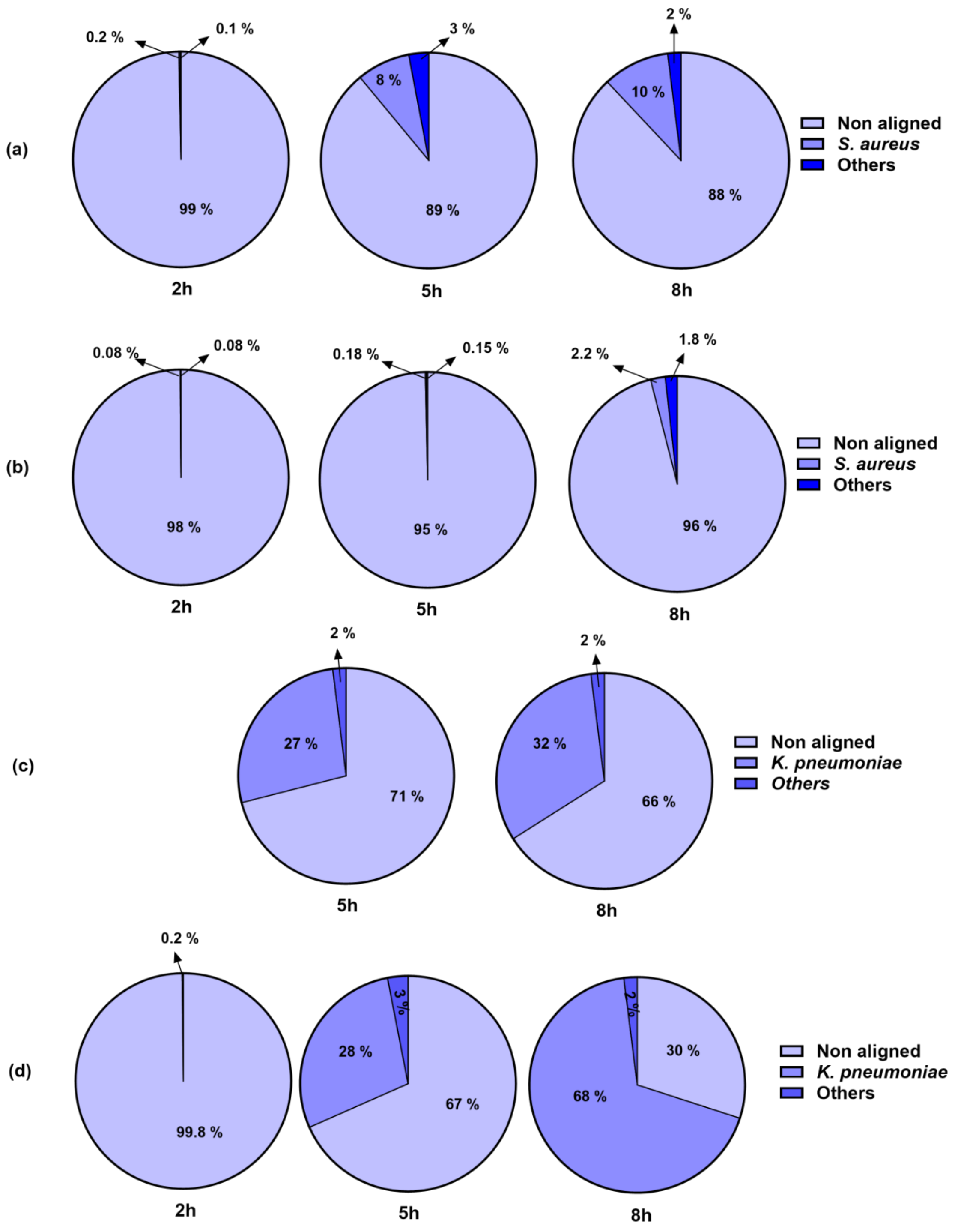

Supplementary Figure S1. The percentage of reads assigned to human and bacterial genomes based on the nanopore sequencing of blood cultures at different incubation time points. **(a)** *S. aureus* NCTC8325 **(b)** *S. aureus* CCUG35600 **(c)** *K. pneumoniae* CCUG225T **(d)** *K. pneumoniae* 225. These results are based on a BLAST search of the acquired sequencing data against the NCBI Reference Prokaryotic (RefProk) database, which only contains prokaryotic genomic data. Sequencing reads that did not align with prokaryotic sequences and were of human origin was designated as "Non-aligned". Additionally, any prokaryotic sequences comprising <1% of the total reads were categorized as "Others".

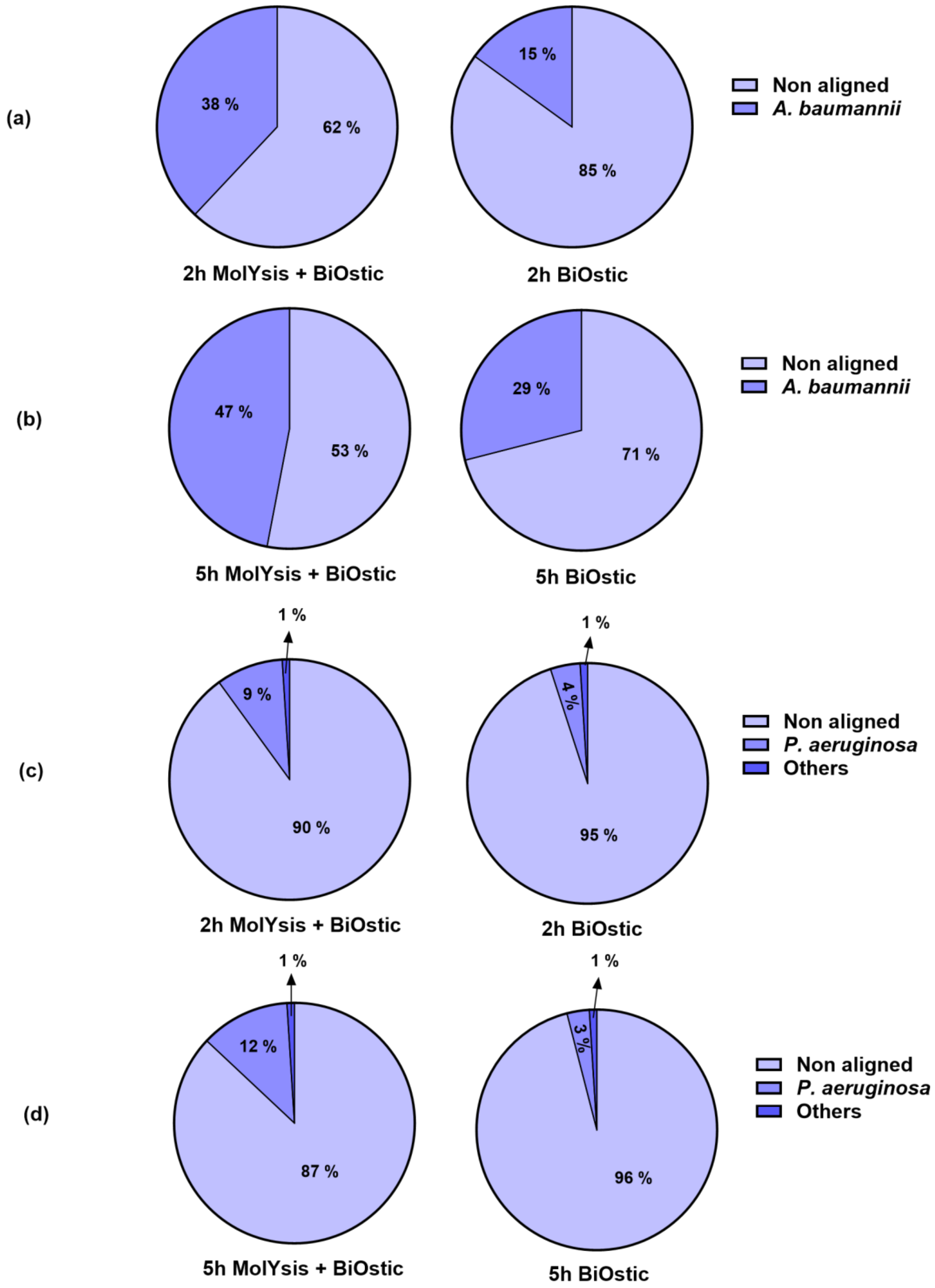

Supplementary Figure S2. Percentage of reads assigned to the human and bacterial genome by BLAST search of the raw nanopore sequencing data against NCBI RefProk database **(a)** 2 hours incubated *A. baumannii* **(b)** 5 hours incubated *A. baumannii* **(c)** 2 hours incubated *P. aeruginosa* **(d)** 5 hours incubated *P. aeruginosa*.

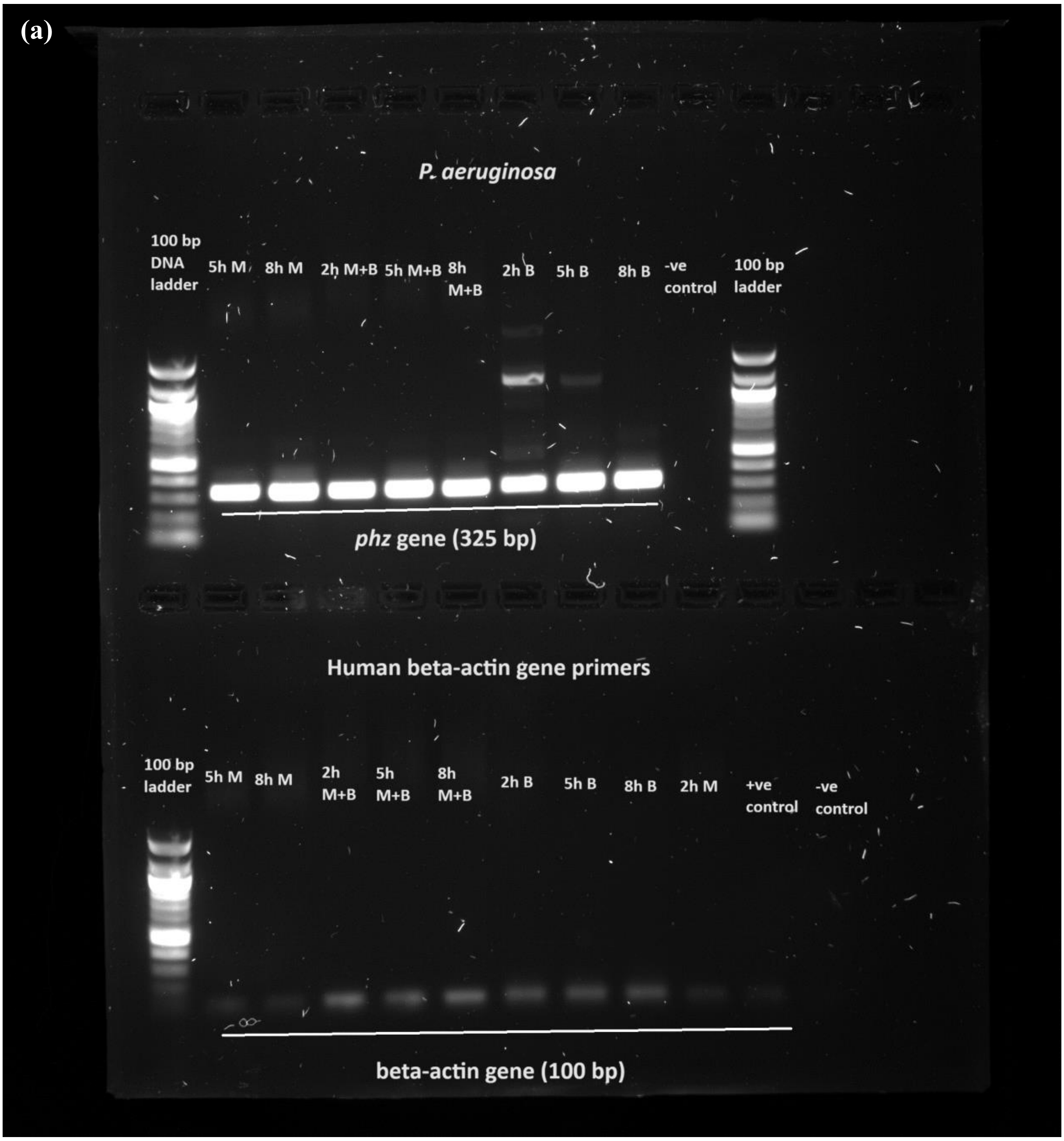

(a)
P. aeruginosa
100 bp DNA ladder
5h M
8h M
2h M+B
5h M+B
8h M+B
2h B
5h B
8h B
-ve control
100 bp ladder
phz gene (325 bp)
Human beta-actin gene primers
100 bp ladder
5h M
8h M
2h M+B
5h M+B
8h M+B
2h B
5h B
8h B
2h M
+ve control
-ve control
beta-actin gene (100 bp)

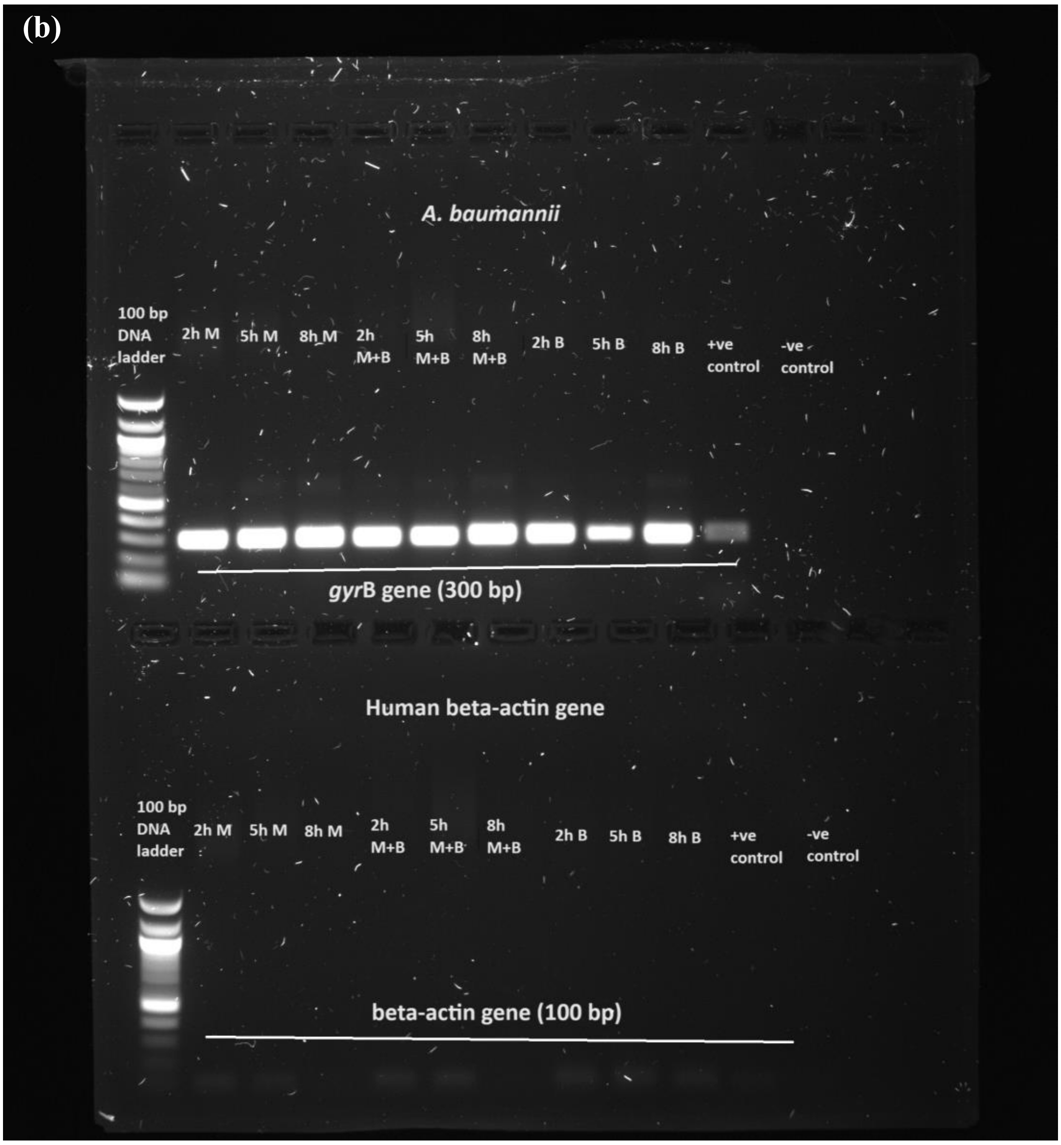

Supplementary Figure S3. PCR results of the DNA extracted using MolYsis complete5, BiOstic bacteremia and a combination of both. **(a)** *P. aeruginosa* (bacterial primers + human primers) **(b)** *A. baumannii* (bacterial primers + human primers). M= MolYsis, B=BiOstic

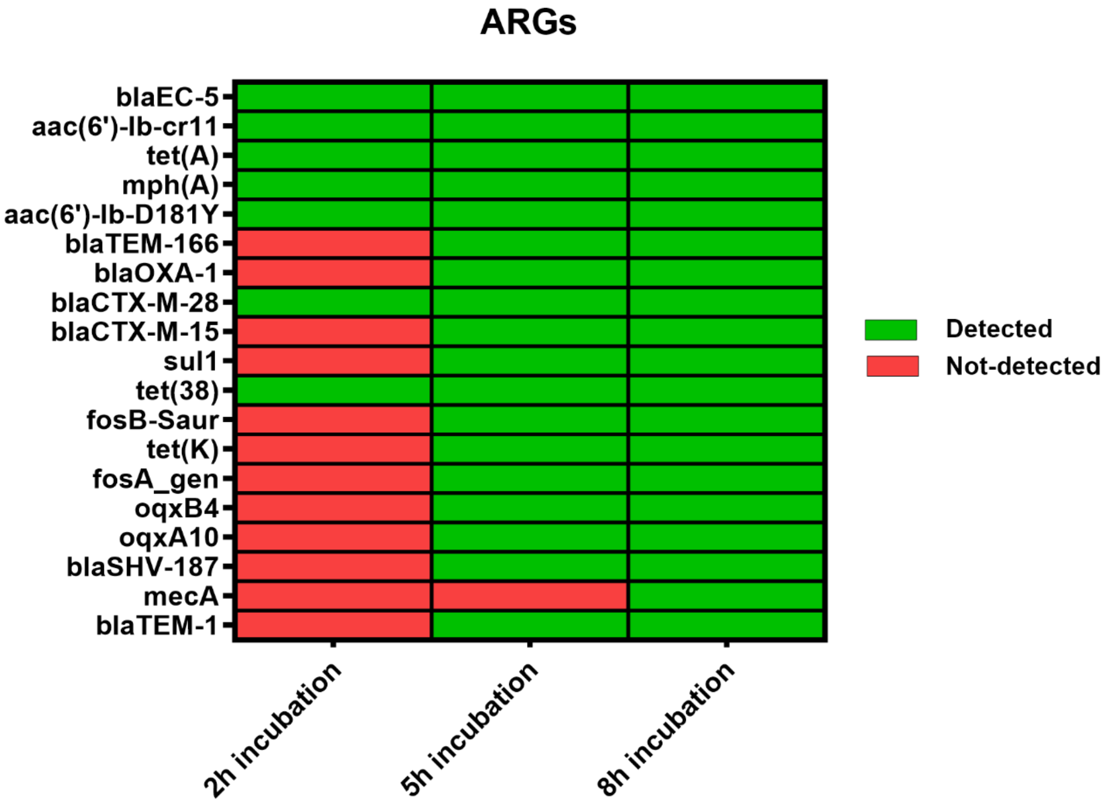

Supplementary Figure S4. Detected ARGs from blood cultures at different time points of incubation using nanopore sequencing.

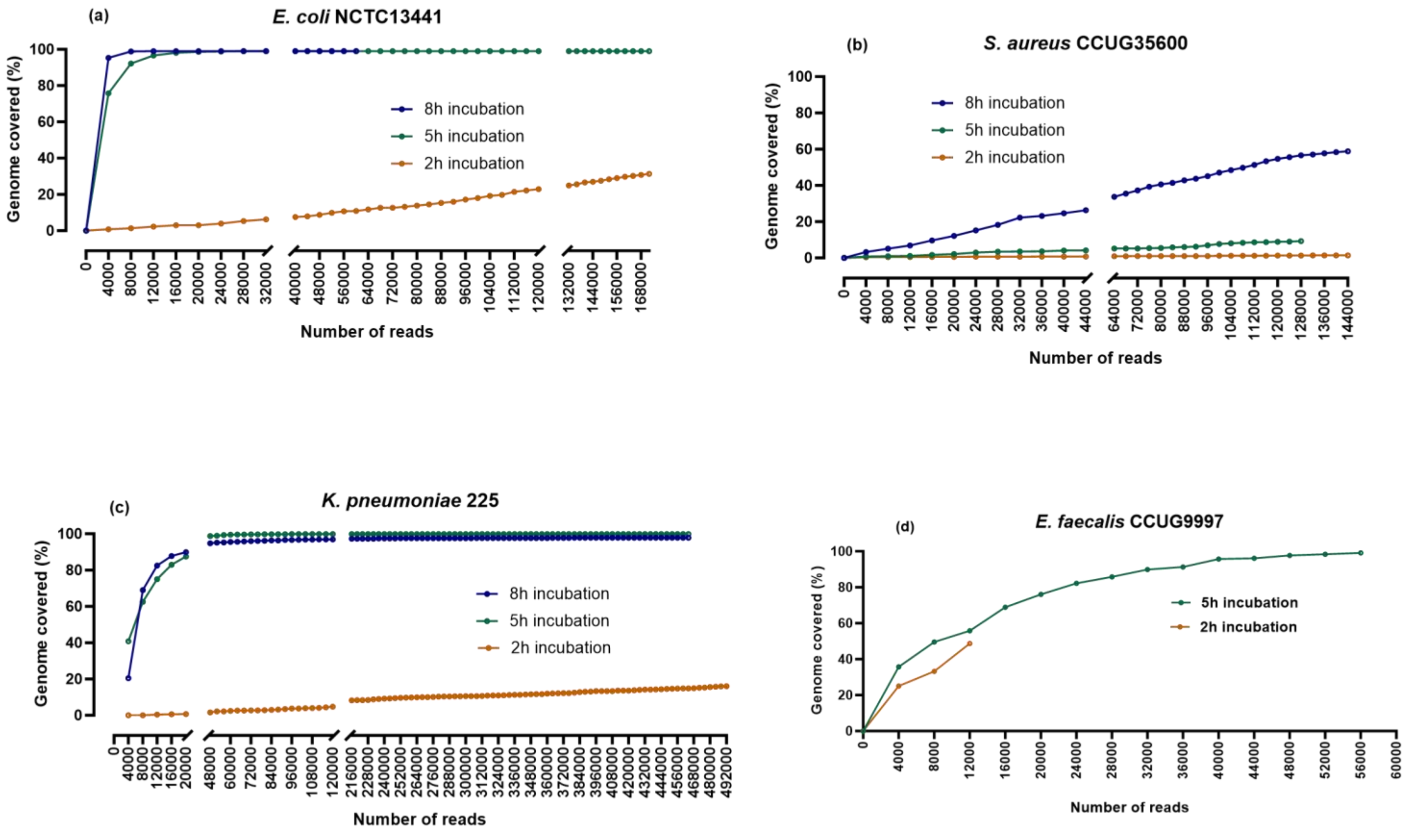

Supplementary Figure S5. Genome coverage of bacterial isolates at different incubation times **(a)** *E. coli* NCTC13441 **(b)** *S. aureus* CCUG35600 **(c)** *K. pneumoniae* 225 **(d)** *E. faecalis* CCUG9997.

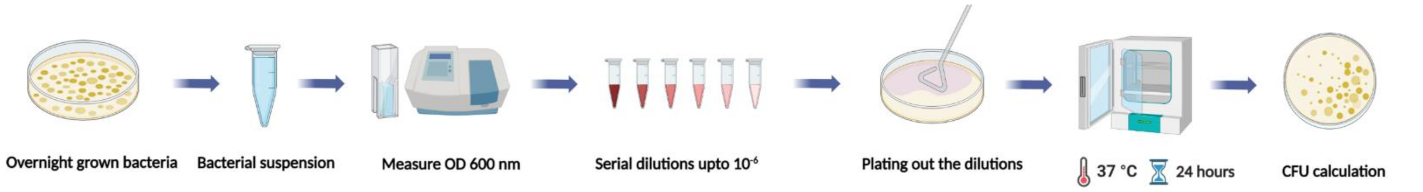

Supplementary Figure S6. An overview of the different steps in optimizing the desired inoculum size (≤ 50CFU/mL).

Supplementary Table S1. Time “0” calculation. Bacterial CFU/mL after each 30 minutes of incubation. *240 minutes was taken as t_0.

		Time in minutes										
**Isolates**		**0**	**30**	**60**	**90**	**120**	**150**	**180**	**210**	**240***	**270**	**300**
E. coli CCUG17620	CFU/mL	0	0	5	8	10	20	40	90	250	600	1400
E. coli NCTC13441		0	0	0	0	0	0	0	4	15	50	150
S. aureus NCTC8325		0	0	0	0	0	0	0	5	30	90	700
S. aureus CCUG35600		0	0	0	0	0	0	0	5	20	60	250
K. pneumoniae CCUG225T		0	0	0	0	0	0	0	5	35	130	850
K. pneumoniae 225		0	0	3	5	8	12	20	50	130	320	850
A. baumannii CCUG19096T		0	2	3	5	7	12	20	40	90	180	380
P. aeruginosa CCUG17619		0	0	0	0	0	0	0	2	6	12	30
E. faecalis CCUG9997		0	3	6	20	55	120	250	820	1550	3500	7700

Supplementary Table S2. The overall time for the detection of bacterial samples from blood cultures at different time points of incubation

					Sequencing Run Time		Overall Time from clinically relevant sepsis sample (Time 0)				
**Bacterial strain**	**AMR gene reference**	**Incubation time (h)**	**CFU/ml**	**DNA (ng/µl)**	**Bacterial ID (mins)**	**ARG (s) detection (mins)**	**Bacterial ID (mins)**	**ARG (s) detection (mins)**	**Total reads**	**Bacterial reads**	**Host reads**
E. coli CCUG17620	*bla*EC-5										
		2	6.6×10^3	97.1	20	60	260	300	389046	56651	328458
		5	5.7×10^6	110	20	20	440	440	270597	171744	98853
		8	2.1×10^9	110	10	10	610	610	267050	204318	62732
E. coli NCTC13441	*CTX-M*-15	2	1.9×10^3	67.4	30	Not detected	270	Not detected	140343	2755	135951
		5	2.6×10^6	110	35	35	455	455	553018	359255	193763
		8	3.5×10^8	110	25	25	625	625	199074	172059	27537
S. aureus NCTC8325	*fos*B	2	1.2×10^4	100	30	Not detected	270	Not detected	400477	822	399655
		5	5×10^6	100	25	95	445	515	478759	56326	410705
		8	3.1×10^7	100	43	43	643	643	132175	15442	115352
S. aureus CCUG35600	*mec*A	2	4.2×10^3	100	40	Not detected	280	Not detected	435829	19305	405870
		5	1.1×10^5	110	45	Not detected	465	Not detected	543377	28682	500119
		8	4.9×10^6	100	40	180	640	780	142050	5837	134579
K. pneumoniae CCUG 225T	*fos*A, *bla*SHV-164	2	1.2×10^3	100	26	Not detected	266	Not detected	565679	525	565154
		5	2×10^6	100	29	45	449	465	116115	33728	82387
		8	1.9×10^7	110	63	129	663	729	102713	34919	67794
K. pneumoniae 225	*bla*SHV-187	2	4.4×10^3	55.3	22	Not detected	262	Not detected	394986	696	394290

		5	1.1×10^{7}	100	29	62	449	482	440642	137424	296718
		8	1×10^{9}	110	24	24	624	624	647638	450282	197356
P. aeruginosa CCUG17619	*bla*OXA-396	2	1.8×10^{2}	110	39	39	279	279	18353	841	17512
		5	9×10^{3}	110	32	32	452	452	21278	962	20316
		8	3×10^{6}	97.3	27	27	627	627	7277	1453	5824
A. baumannii CCUG19096T	*bla*ADC-158, *sul*2	2	1.6×10^{3}	100	35	35	275	275	16910	2560	14350
		5	1.6×10^{4}	100	27	27	447	447	11078	3230	7848
		8	2.4×10^{7}	96.9	22	22	622	622	8028	3518	4510
E. faecalis CCUG9997	*tet*M	2	4×10^{4}	74.8	25	Not detected	265	Not detected	14626	3117	11509
		5	1×10^{7}	116	20	20	440	440	147661	102214	45447
		8	1.2×10^{9}	120	Not sequenced						

Supplementary Table S3. List of the primers used for species-specific PCR amplification.

Target species	Target gene	Product size	Primer	Primer's sequence
**Human**	β-Actin	~ 100 bp	β-Actin-F	CGGCCTTGGAGTGTGTATTAAGTA
			β-Actin-R	TGCAAAGAACACGGCTAAGTGT
P. aeruginosa	*phz*A2	~ 325 bp	*phz*A2-F	GTTTACCGACAACCTGGAA
			*phz*A2-R	GCAATAGCCCTGCGGATAC
A. baumannii	*gyr*B	~ 300 bp	*gyr*B-F	CACGCCGTAAGAGTGCATTA
			*gyr*B-R	AACGGAGCTTGTCAGGGTTA

Supplementary Table S4. Overview of the bacterial isolates, phenotype, and reference AMR genes.

S. No	Isolate	Gram staining	Phenotype based on AST (antibiotic) wild type (WT)/non-wild type (NWT)	Reference antibiotic resistance gene (s)
1	*E. coli* CCUG17620	Negative	Susceptible-WT	*bla*EC-5
2	*E. coli* NCTC13441	Negative	Resistant-NWT	*bla*TEM-1, *bla*CTX-M-15
3	*S. aureus* NCTC8325	Positive	Susceptible-WT	*fos*B
4	*S. aureus* CCUG35600	Positive	Resistant-NWT	*mec*A
5	*K. pneumoniae* CCUG225T	Negative	Susceptible-WT	*bla*SHV-164, *fos*A
6	*K. pneumoniae* 225	Negative	Resistant-NWT	*bla*SHV-187
7	*A. baumannii* CCUG19096T	Negative	Susceptible-WT	*sul*2, *bla*ADC-158
8	*P. aeruginosa* CCUG17619	Negative	Susceptible-WT	*blaOXA*-396
9	*E. faecalis* CCUG9997	Positive	Resistant-NWT	*tet*M

Supplementary Table S5. Correlation of bacterial CFU/mL and absorbance at OD_{600nm}.

Pathogens	A600 nm	CFU/mL
E. coli CCUG17620	0.15	7.8×10^{6}
	0.24	2.3×10^{7}
	0.306	6.6×10^{7}
	1.20	7.1×10^{8}
	1.49	1.4×10^{9}
	1.53	1.1×10^{9}
	1.71	2.1×10^{9}
	1.78	1.2×10^{9}
E. coli NCTC13441	0.16	1.3×10^{7}
	0.26	3.4×10^{7}
	1.31	6.1×10^{8}
	1.39	8.0×10^{8}
S. aureus NCTC8325	0.18	1.3×10^{6}
	0.20	3.4×10^{6}
	0.35	2.2×10^{7}
	1.38	7.9×10^{8}
S. aureus CCUG35600	0.17	8.6×10^{5}
	0.20	1.1×10^{6}
	1.28	1.0×10^{9}
	1.59	1.7×10^{9}
K. pneumoniae CCUG225T	0.16	2.5×10^{6}
	0.19	7.6×10^{6}
K. pneumoniae 225	0.13	3.2×10^{6}
	0.16	5.6×10^{6}
P. aeruginosa CCUG17619	0.86	1.4×10^{8}
	1.56	2.0×10^{8}
	1.9	2.4×10^{9}
A. baumannii CCUG19096T	0.88	3.0×10^{8}
	1.03	4.2×10^{8}
	1. 45	5.8×10^{7}
E. faecalis CCUG9997	1.8	1×10^{9}
	0.7	3.2×10^{7}
	0.4	1×10^{5}
